# MAP4K4 exacerbates cardiac microvascular injury in diabetes by facilitating S-nitrosylation modification of Drp1

**DOI:** 10.1186/s12933-024-02254-7

**Published:** 2024-05-09

**Authors:** Yuqiong Chen, Su Li, Bo Guan, Xiaopei Yan, Chao Huang, Yingqiang Du, Fan Yang, Nannan Zhang, Yafei Li, Jian Lu, Jiankang Wang, Jun Zhang, Zhangwei Chen, Chao Chen, Xiangqing Kong

**Affiliations:** 1 Department of Cardiology, Gusu School, The Affiliated Suzhou Hospital of Nanjing Medical University, Suzhou Municipal Hospital, Nanjing Medical University, 215000 Suzhou, Jiangsu Province China; 2 Department of Cardiology, Shanghai Institute of Cardiovascular Diseases, Zhongshan Hospital, Fudan University, 180 Fenglin Road, 200032 Shanghai, China; 3 Department of Geriatrics, Gusu School, The Affiliated Suzhou Hospital of Nanjing Medical University, Suzhou Municipal Hospital, Nanjing Medical University, Suzhou, China; 4 Department of Respiratory Medicine, Gusu School, The Affiliated Suzhou Hospital of Nanjing Medical University, Suzhou Municipal Hospital, Nanjing Medical University, Suzhou, China; 5 Ministry of Science and Technology, the Affiliated Suzhou Hospital of Nanjing Medical University, Suzhou Municipal Hospital, 215002 Suzhou, Jiangsu China; 6 Department of Endocrinology, Endocrine and Metabolic Disease Medical Center, Affiliated Hospital of Medical School, Nanjing Drum Tower Hospital, Nanjing University, 210008 Nanjing, China; 7 Branch of National Clinical Research Center for Metabolic Diseases, 210008 Nanjing, China; 8 Department of Critical Care Medicine, the Affiliated Suzhou Hospital of Nanjing Medical University, Suzhou, China; 9 Department of Cardiology, Gulou District, the First Affiliated Hospital of Nanjing Medical University, 300 Guangzhou Road, Nanjing City, Jiangsu Province China

**Keywords:** MAP4K4, S-nitrosylation, Drp1, Cardiac microvascular injury, Diabetic cardiomyopathy

## Abstract

Dynamin-related protein 1 (Drp1) is a crucial regulator of mitochondrial dynamics, the overactivation of which can lead to cardiovascular disease. Multiple distinct posttranscriptional modifications of Drp1 have been reported, among which S-nitrosylation was recently introduced. However, the detailed regulatory mechanism of S-nitrosylation of Drp1 (SNO-Drp1) in cardiac microvascular dysfunction in diabetes remains elusive. The present study revealed that mitogen-activated protein kinase kinase kinase kinase 4 (MAP4K4) was consistently upregulated in diabetic cardiomyopathy (DCM) and promoted SNO-Drp1 in cardiac microvascular endothelial cells (CMECs), which in turn led to mitochondrial dysfunction and cardiac microvascular disorder. Further studies confirmed that MAP4K4 promoted SNO-Drp1 at human C644 (mouse C650) by inhibiting glutathione peroxidase 4 (GPX4) expression, through which MAP4K4 stimulated endothelial ferroptosis in diabetes. In contrast, inhibition of MAP4K4 via DMX-5804 significantly reduced endothelial ferroptosis, alleviated cardiac microvascular dysfunction and improved cardiac dysfunction in db/db mice by reducing SNO-Drp1. In parallel, the C650A mutation in mice abolished SNO-Drp1 and the role of Drp1 in promoting cardiac microvascular disorder and cardiac dysfunction. In conclusion, our findings demonstrate that MAP4K4 plays an important role in endothelial dysfunction in DCM and reveal that SNO-Drp1 and ferroptosis activation may act as downstream targets, representing potential therapeutic targets for DCM.

**Supplementary Information:**

The online version contains supplementary material available at 10.1186/s12933-024-02254-7.

## Introduction

Diabetic cardiomyopathy (DCM) is a prominent complication of type 2 diabetes mellitus [1]. The main pathological characteristic features of DCM are cardiac systolic and diastolic dysfunction, which are mainly induced by myocardial hypertrophy and myocardial interstitial fibrosis [2, 3]. Cardiac microvascular endothelial cells (CMECs) are essential components of heart tissues and are crucial for guaranteeing appropriate microvascular perfusion [4, 5]. A high-glucose environment and lipid metabolism disorders cause chronic impairment of CMECs and microcirculatory dysfunction and in turn exacerbate cardiac dysfunction and myocardial pathological remodeling [6, 7]. However, the specific molecular mechanism underlying endothelial dysfunction in DCM remains unclear.

Mitogen-activated protein kinase kinase kinase kinase 4 (MAP4K4), also called Nck-interacting kinase (NIK), is a member of the Ste20 protein kinase family [8]. MAP4K4 has been widely reported to be a serine/threonine kinase that is closely related to diverse physiological and pathophysiological processes, such as normal embryonic development, atherosclerosis, insulin sensitivity and inflammatory reactions [9, 10]. Study has shown that endothelial-specific MAP4K4 knockout causes postnatal lethality, highlighting the pivotal role of MAP4K4 in vascular development and homeostasis [11]. Moreover, MAP4K4 has been suggested to promote angiogenesis under pathological conditions [12]. Conversely, research has shown that silencing MAP4K4 contributes significantly to promote aortic endothelial cell (EC) activation and improve endothelial permeability [13]. This discrepancy makes it difficult to identify the regulatory effect of MAP4K4 on cardiac microcirculation in DCM.

Mitochondrial dynamics play important roles in signal transduction and mitochondrial quality control [14, 15]. Studies have revealed that mitochondrial dynamics disorders play a crucial role in the occurrence and progression of coronary microvascular disorders in DCM patients [14, 16]. Increased mitochondrial fragmentation has been observed in venous ECs from diabetic patients [17]. In line with clinical observations, aberrant mitochondrial dynamics in CMECs were found in animal experiments and have been attributed to the pathogenesis of cardiac microvascular disorders. Pathological mitochondrial fission disrupts coronary endothelium-dependent relaxation due to mitochondrial ROS production [18]. In addition, diabetes-induced Drp1-dependent mitochondrial fission promotes mitochondrial apoptosis in CMECs, thus contributing to capillary degeneration in diabetes [19]. Moreover, mitochondrial fission impairs vascular permeability, migration, and angiogenesis in DCM [19]. Drp-1 serves as a critical effector of mitochondrial fission in diabetes [20]. Phosphorylation and mitochondrial translocation are the classical indices of Drp1 activation, and these processes are believed to be under the control of posttranslational modifications, such as S-nitrosylation, ubiquitination and SUMOylation [21, 22]. Among these modifications, the S-nitrosylation of Drp1 (SNO-Drp1) is associated with various neuronal diseases [23]. Only limited evidence has established a link between SNO-Drp1 and cardiovascular diseases [24]. Whether S-nitrosylation of Drp-1 occurs in diabetes and contributes to cardiac microvascular disorders merits further investigation.

In recent years, emerging evidence has indicated that ferroptosis, a nonapoptotic cell death pattern characterized by iron overload and lipid hydroperoxides, plays a pathophysiological role in the development of DCM [25]. Diabetes promotes ferroptosis in cardiomyocytes and causes cardiac diastolic dysfunction, which can be reversed by activating the AMPK-NRF2 pathway [26]. Mitochondrial damage is another feature of ferroptosis. Abnormal mitochondrial structure, rupture of the mitochondrial outer membrane, and reduced mitochondrial membrane potential were observed in the hearts of diabetic mice [27]. Diabetes promotes endothelial ferroptosis and cardiac microvascular injury in DCM. Our recent study demonstrated that diabetes causes mitochondrial dysfunction and excites mitochondrial iron overload and lipid hydroperoxides in CMECs, whereas maintaining mitochondrial dynamics via MFN2 inhibited endothelial ferroptosis by suppressing the mitochondrial translocation of ACSL4 [6]. Moreover, the elimination of mitochondrial fragments via AMPKα1-Parkin pathway-dependent mitophagy has been shown to alleviate endothelial ferroptosis and cardiac microvascular disorders in DCM [7]. However, whether Drp1 and S-nitrosylated Drp1 exacerbate endothelial ferroptosis in DCM remains unknown.

Here, our data demonstrated that MAP4K4 plays a crucial role in microcirculatory disturbance in DCM. In diabetes, upregulated MAP4K4 dramatically interferes with mitochondrial morphology and function, inhibits GPX4 expression, and promotes endothelial ferroptosis by stimulating SNO-Drp1, ultimately promoting cardiac microvascular injury. Thus, our data identify MAP4K4 as a key regulator of SNO-Drp1, which is a potential therapeutic target for DCM in diabetes.

## Materials and methods

### Animals

Adult male db/db mice and littermate nondiabetic male db/m mice were purchased from Shanghai SLAC Laboratory Animal Co., Ltd. (China). Throughout the experiment, the mice were kept in standard housing with free access to food and drinking water. All animal experiments were ethically approved by the Animal Experimental Ethics Committee and performed according to the animal experiment guidelines of Nanjing Medical University and Fudan University.

Four-week-old male db/m and db/db mice were transfected with Tie2-enhanced adeno-associated virus (AAV9) carrying shMAP4K4, wild-type Drp1 (Drp1-WT) or Cys650-mutated Drp1 (Drp1-C650A) via the tail vein to achieve EC-specific overexpression or knockdown of these proteins, and a total of 6 × 10^11^ vector genomes were injected into db/db mice via the tail vein every 8 weeks for 24 weeks. The EC transfection efficiency and specificity of AAV9 were confirmed by western blotting and immunofluorescence. For MAP4K4 inhibitor treatments, DMX-5804 (MCE, USA) was orally administered at a dose of 3 mg/kg three times per week at 4 weeks of age for 24 weeks [28].

### CMEC culture and treatment

The extraction and culture of primary CMECs from cardiac samples were performed as previously described [2]. After dissecting the coronary arteries, endocardium and epicardium, ventricular tissue was harvested to prepare cell suspensions. Then, anti-CD31 magnetic beads (Thermo Fisher Scientific) were added and incubated with the cell suspension at 4 °C with low-speed rotation for 30 min. The primary CMECs were purified by magnetic isolation and then seeded in confocal dishes for fluorescence imaging or lysed for protein extraction.

For in vitro experiments, human CMECs (HCMECs, Lonza Bioscience) were incubated and cultured in EC medium (ECM, ScienCell Research Laboratories). HCMECs were cultured to 90% confluence before treatment with high glucose (HG, 25 mmol/L) and free fatty acids (FFAs, 0.5 mmol/L) for 72 h. The mixed FFA solutions were prepared as described previously [6]. CMECs were transfected with lentiviruses (LVs) encoding shMAP4K4, MAP4K4, PDI, CBR1, GPX4, shGPX4 or their negative controls at the appropriate multiplicity of infection (MOI) according to the manufacturers’ instructions. The transfection efficiencies were confirmed by western blot analysis. For in vitro testing, the MAP4K4 inhibitor DMX-5804 (5–15 μM) or L-NAME (50 μM, MCE, USA) was used for 72 h [29].

Genomic mutations were introduced into cells using the CRISPR–Cas9 system. Single-guide RNAs (sgRNAs) were designed by Genomeditech (China, Shanghai) to target the genomic area adjacent to mutation sites in C505 or C644. After reaching 60% confluence, the cells were cotransfected with sgRNAs (0.5 μg) and single-stranded donor oligonucleotides (ssODNs) as templates to introduce mutations. Twenty-four hours after transfection, the cells were trypsinized, diluted to obtain single cells and seeded into 96-well plates. Genomic DNA was extracted, followed by sequencing of the PCR products spanning the mutation sites.

### Echocardiography

Cardiac function was evaluated by two-dimensional echocardiography. The left ventricular ejection fraction (LVEF), left ventricular fractional shortening (LVFS), left ventricular end-diastolic dimension (LVEDD), and E/A ratio were calculated. After the echocardiography, the mice were humanely euthanized for serum and myocardial tissue collection. Heart weight and tibia length were measured to calculate the heart hypertrophy index.

### Histopathologic staining

The cardiac tissues were fixed in 4% paraformaldehyde, dehydrated, embedded in paraffin, and sectioned into 4-mm sections. Myocardial fibrosis and collagen content were evaluated by Masson’s trichrome staining, and cardiac hypertrophy was assessed using wheat germ agglutinin (WGA) staining. The degree of fibrosis and the cross-sectional area of the cardiomyocytes were calculated using ImageJ software (version 1.53c, NIH, USA).

### Fluorescence staining of cells and tissues

For myocardial tissue microvascular perfusion, 100 μL of FITC-conjugated lectin (1 mg/mL) was injected intravenously into the mice through the tail vein to assess myocardial perfusion [30, 31]. Ten minutes after lectin injection, the mouse heart samples were frozen, made into 5 μm sections, and further processed through immunostaining with an anti-CD31 antibody. The perfused microvascular density is expressed as the ratio of lectin-FITC-labeled microvessels to CD31-highlighted microvessels.

To observe mitochondrial morphology, CMEC mitochondria were stained with MitoTracker Red (Invitrogen) according to the manufacturer’s instructions. Total intracellular reactive oxygen species (ROS) and mitochondrial ROS (mitoROS) generation were detected using a Reactive Oxygen Species Assay Kit (Beyotime, China) and mitoSOX superoxide indicator (Invitrogen), respectively, after 30 min of incubation. Fluorescence images were obtained using a laser confocal microscope (FV3000, Olympus, Japan) and qualified using ImageJ software.

### Cell viability and LDH detection

Cell viability was examined using a Cell Counting Kit-8 (CCK-8, Epizyme, China) based on the manufacturer’s instructions. Cells were seeded in 96-well plates prior to the addition of CCK-8 working solution (100 μL) to each well. Then, the plates were incubated for 4 h at 37 °C in the dark. The absorbance was read at 450 nm by a plate reader (Thermo Scientific, USA). LDH release was measured using an LDH Cytotoxicity Assay Kit (Beyotime) according to the manufacturer’s instructions, and the absorbance was detected at 490 nm.

### NO content

Nitric oxide (NO) content in myocardial tissues and NO release from CMECs were assessed using an NO assay kit (Beyotime). For measurement of NO release from CMECs, the cell culture medium was directly assessed according to the manufacturer’s instructions, and the value was normalized to the cell number (5 × 10^5^). For myocardial tissue, the samples were homogenized and centrifuged (12,000×*g*, 15 min) to collect the supernatant. The protein concentration was quantified using a Bradford protein assay kit (Solarbio, China). Then, the NO content in cardiac tissue was normalized to the protein concentration.

### Tissue and cellular monolayer permeability measurements

Heart tissue permeability was quantified by the Evans blue (EB) method according to previously reported protocol [32]. Mice were administered EB dye (10 mg/ml) via the tail vein for 30 min before sacrifice. Then, the hearts were removed, dissected, and weighed. Afterward, the samples were placed into formamide solution (500 μl), fully ground, incubated at 65–70 °C overnight, and then centrifuged at 10,000×*g* for 40 min to collect the supernatant. The absorbance of the supernatant and standards was measured using a plate reader (Thermo Scientific, USA) at 620 nm.

To evaluate cellular monolayer permeability in vitro, ten thousand HCMECs were inoculated in the upper chamber of a Transwell insert (0.4 μm pore size, Corning, USA) for 3 days to confirm stable monolayer junction formation. Then, 1 mg/mL FITC-dextran (100 μL, Solarbio) was added to the upper chamber. The amount of FITC-dextran that penetrated the lower chamber through the paracellular space was determined according to the FITC-dextran fluorescence intensities [2].

Transendothelial electrical resistance (TEER) was measured to assess junctional function. CMECs were seeded onto fibronectin-coated inserts. After the cells reached confluence and were subjected to the above treatments, a MilliCell ERS-2 system (Millipore, USA) was used to measure the TEER [19].

### Assessment of mitochondrial function

Mitochondrial respiration activity was measured according to oxygen consumption rates (OCRs), which were assessed via a Seahorse XF96 Extracellular Flux Analyzer (Seahorse Bioscience, USA) as reported previously [33]. HCEMCs were seeded into XF24 cell culture plates at 5 × 10^4^ cells/well. Following HG/FFA exposure, the HCMECs were serum starved for 6 h, and then oligomycin, FCCP, rotenone, and antimycin A were sequentially added during the assay according to the manufacturer’s protocols to determine the OCR profiles. The basal OCR, ATP-linked OCR, maximal OCR and spare respiratory OCR were calculated as previously described [34].

For detection of the mitochondrial membrane potential (MMP), CMECs were seeded in a 96-well black plate (Corning, USA) and incubated with 5 μM TMRM (Invitrogen, USA) at 37 °C for 20 min in the dark. The MMP was calculated as the fluorescence intensity (red) divided by the total cell number.

Coenzyme Q10 (CoQ10) concentrations were detected via a CoQ10 ELISA Kit (CUSABIO, China) according to the product instructions. After treatment, the HCMECs were harvested, homogenized, and centrifuged (10,000×*g*, 10 min) to collect the supernatants. The obtained supernatant was mixed and incubated with HRP-conjugate at 37 °C for 40 min. Following sufficient washing, TMB substrate was added, and the mixture was incubated for 25 min at 37 °C in the dark. After adding the termination solution, the absorbance values of each group were obtained at 450 nm.

### Lipid peroxidation and ROS production

The lipid peroxide (LPO) content and MDA level were detected with an LPO Content Assay Kit (Solarbio) and an MDA assay kit (Beyotime), respectively, following the manufacturer’s instructions. The protein contents of the sample lysates were quantified by a BCA assay immediately after the intervention. The LPO and MDA levels were calculated according to the basis of total protein content. Iron levels were examined with an Iron Assay Kit (Abcam, USA). ROS levels were measured using a reactive oxygen species assay kit (Nanjing Jiancheng Bioengineering Institute, China), and H_2_O_2_ content was evaluated by Amplex Red (Beyotime). All the above experiments were performed according to the manufacturers’ instructions.

### Transwell assay

CMECs were collected in serum-free medium and seeded in the upper chambers of Corning Transwell chambers (8 µm pores). ECM containing 10% serum was added to the lower compartment to induce cell migration. After 24 h of migration, the migrated cells were fixed with 4% PFA and stained with crystal violet (Solarbio). For each sample, five microscopic visual fields were randomly selected, and the average cell number of these fields was calculated with ImageJ (version 1.53c, NIH, USA).

### RNA extraction and real-time PCR

Prior to PCR, total RNA was extracted using a Total RNA Extraction Kit (Solarbio) according to the manufacturer’s instructions. Then, the isolated mRNA was treated with DNase I (Takara, Japan) and reverse-transcribed into cDNA using a cDNA reverse transcription kit (Invitrogen, USA). Real-time quantitative PCR was carried out using SYBR Green I (TSINGKE, China) on a Bio-Rad CFX96 Real-Time PCR system. The primers used are listed in Additional file 6: Table S1. The results of qPCR are shown as the 2^−ΔΔCt^ method.

### Biotin switch assay of S-nitrosylated proteins

A biotin-switch assay was carried out using an S-nitrosylated protein detection kit (Cayman Chemical, USA) as previously described [35]. Briefly, whole-cell lysates, cytosolic fractions and mitochondria were collected and incubated in blocking reagent. Thereafter, the supernatant was collected and incubated with ice-cold acetone at − 20 °C. The reduction of S-nitrosothiol groups was carried out with reducing buffer to yield free thiols. Thereafter, the samples were covalently labeled with biotin in labeling buffer, and the biotinylated proteins were purified by streptavidin-coupling beads with agitation. The S-nitrosylation of Drp1 was quantified by SDS‒PAGE.

### Western blot analysis and immunoprecipitation (IP)

Mitochondria were extracted using a mitochondria isolation kit (Beyotime, China) according to the manufacturer’s instructions. Proteins were extracted using RIPA lysis buffer (Millipore, USA) supplemented with protease inhibitors and then centrifuged at 12,000 rpm (20 min, 4 °C). The protein samples were quantified via a BCA assay, separated via SDS‒PAGE, and transferred electrophoretically onto polyvinylidene difluoride (PVDF) membranes. Then, the membranes were blocked, incubated overnight with primary antibodies at 4 °C, and incubated for 1 h with conjugated secondary antibodies at room temperature. The protein bands were detected with electrochemiluminescence western blotting substrate (Thermo Fisher, USA), and the luminescence signals were measured with ImageJ software. The primary antibodies used are listed in Additional file 6: Table S2.

To perform a coimmunoprecipitation (Co-IP) assay, the cells were lysed using Cell Lysis Buffer for IP (Beyotime) and incubated with Ig-A/G-magnetic beads (BioLinkedIn, China) preconjugated with antibodies against GPX4 (Santa Cruz, sc-166570) at 4 °C overnight. After extensive washing, the immunocomplexes were mixed with loading buffer and boiled for 10 min to elute the target proteins.

### Statistical analysis

The data were analyzed using GraphPad Prism software (version 10.0, GraphPad Software, USA). All the data are presented as the mean ± SEM. Statistical analyses were performed using Student’s t test, one-way ANOVA with Tukey’s post hoc test, and two-way ANOVA with Bonferroni correction. Differences were considered significant when *P < 0.05, **P < 0.01, or ***P < 0.001.

## Results

### Endothelial-specific MAP4K4 knockdown alleviated cardiac microvascular disorders and cardiac dysfunction in DCM

**Fig. 1:**
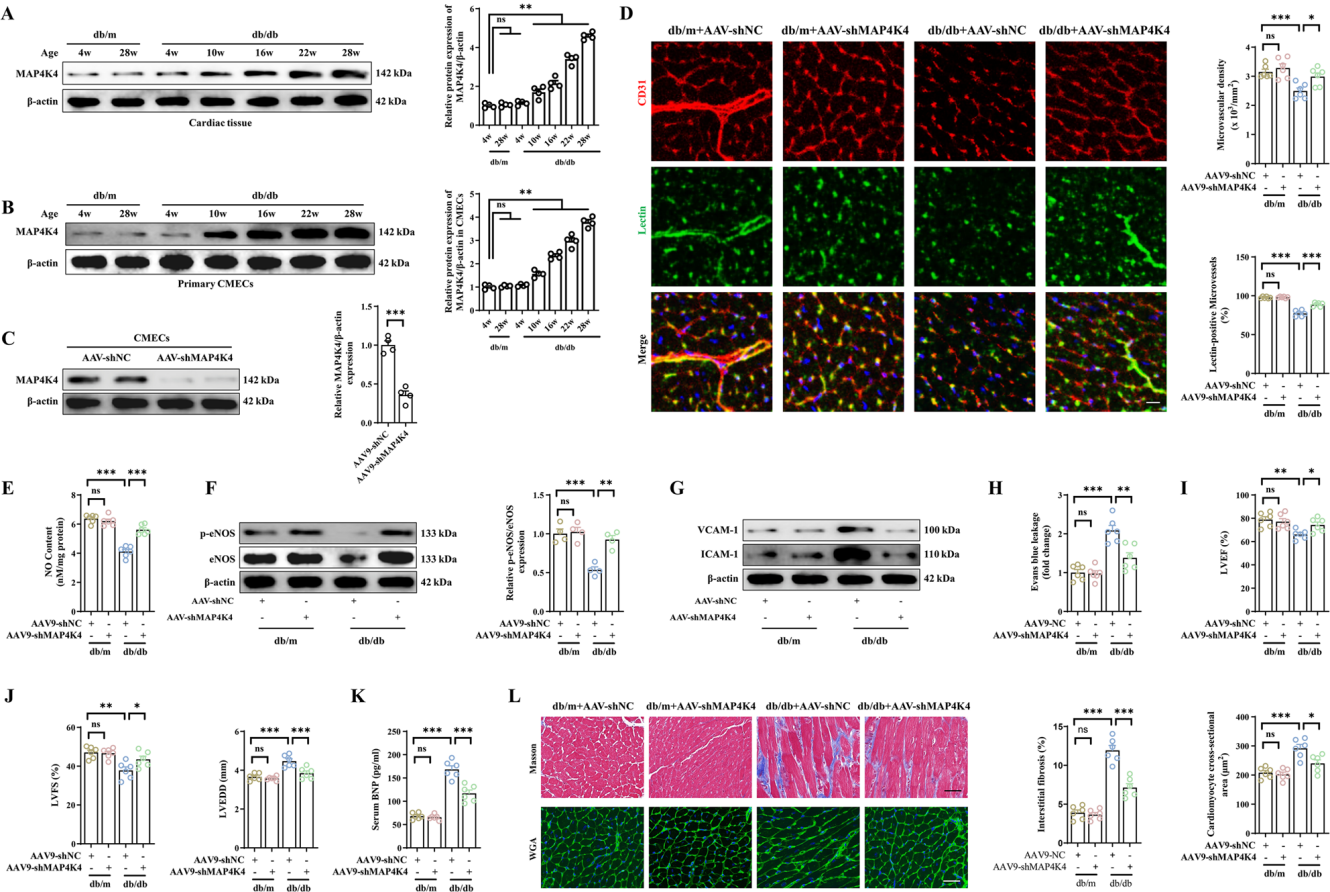
Endothelial-specific knockdown of MAP4K4 ameliorated myocardial microcirculation injury and prevented ventricular remodeling after long-term diabetes. Four-week-old male db/db mice and age-matched db/m mice were transfected with AAV9-shMAP4K4 or AAV9-shNC for 24 weeks. **A–B** Protein expression and quantitative analysis of MAP4K4 in myocardial tissue and primary CMECs from db/m and db/db mice. **C** The transfection efficiency of AAV9-shMAP4K4 was measured by western blotting in primary CMECs. **D** Cardiac microvascular density was assessed by immunofluorescence staining of CD31, and microvascular blood flow was assessed by a lectin-FITC perfusion assay. Scale bar = 25 μm. **E** Quantitative analysis of NO content in myocardial tissues. **F** Total and phosphorylated eNOS protein levels. **G** Protein expression of VCAM-1 and ICAM-1. **H** Quantitative analysis of EB leakage. **I–J** Statistical analysis of the LVEF, LVFS, and LVEDD. **K** Quantitative analysis of serum BNP levels. **L** Cardiac fibrosis and cardiomyocyte cross-sectional area were detected by Masson trichrome staining and WGA staining, respectively. Scale bar = 70 μm. *p < 0.05, **p < 0.01, ***p < 0.001 indicate significant differences. Four to six biological replicates were performed, and the results are indicated in scatter plots

To date, no available studies have assessed the role of MAP4K4 in cardiac microvascular disorders caused by diabetes. Therefore, db/db mice were used as a diabetes model to assess the effects of MAP4K4 on endothelial dysfunction in vivo. Western blot analysis revealed that the expression of MAP4K4 gradually increased over time in both left ventricular tissues and primary CMECs (Fig. 1A–B). Therefore, db/db mice aged 28 weeks were selected as the research objects.

To assess the potential influence of MAP4K4 on diabetic myocardial microcirculation, endothelial-specific deletion of MAP4K4 was induced by AAV-shMAP4K4 transfection (Fig. 1C, Additional file 1: Fig. S1A, B). Lectin perfusion assays revealed that microvascular density and perfusion were evidently reduced in db/db mice, but these effects were obviously alleviated by silencing MAP4K4 (Fig. 1D). Moreover, MAP4K4 knockdown significantly reversed the decreases in NO content and eNOS phosphorylation, suggesting that MAP4K4 improved endothelial-dependent vasodilatation (Fig. 1E, F). Furthermore, the levels of vascular adhesion factors (VCAM-1 and ICAM-1) and vascular leakage were significantly reduced after endothelial-specific knockdown of MAP4K4 in db/db mice (Fig. 1G, H).

Subsequently, we investigated the benefits of MAP4K4 knockdown on cardiac dysfunction and pathological remodeling in DCM. The echocardiography results showed severely decreased cardiac function in db/db mice, as evidenced by decreased left LVEF, LVFS and E/A ratio, as well as increased LVEDD and serum BNP levels (Fig. 1I–K, Fig. S1C). In addition, chronic diabetes exacerbated myocardial interstitial fibrosis and contributed to cardiac hypertrophy in db/db mice (Fig. 1L). In contrast, MAP4K4 silencing significantly improved myocardial systolic and diastolic function and reversed cardiac ventricular remodeling in db/db mice (Fig. 1I–L, Fig. S1C). Overall, the above results demonstrated that the microvascular-protective effects caused by silencing MAP4K4 could protect against diabetes.

**Fig. 2:**
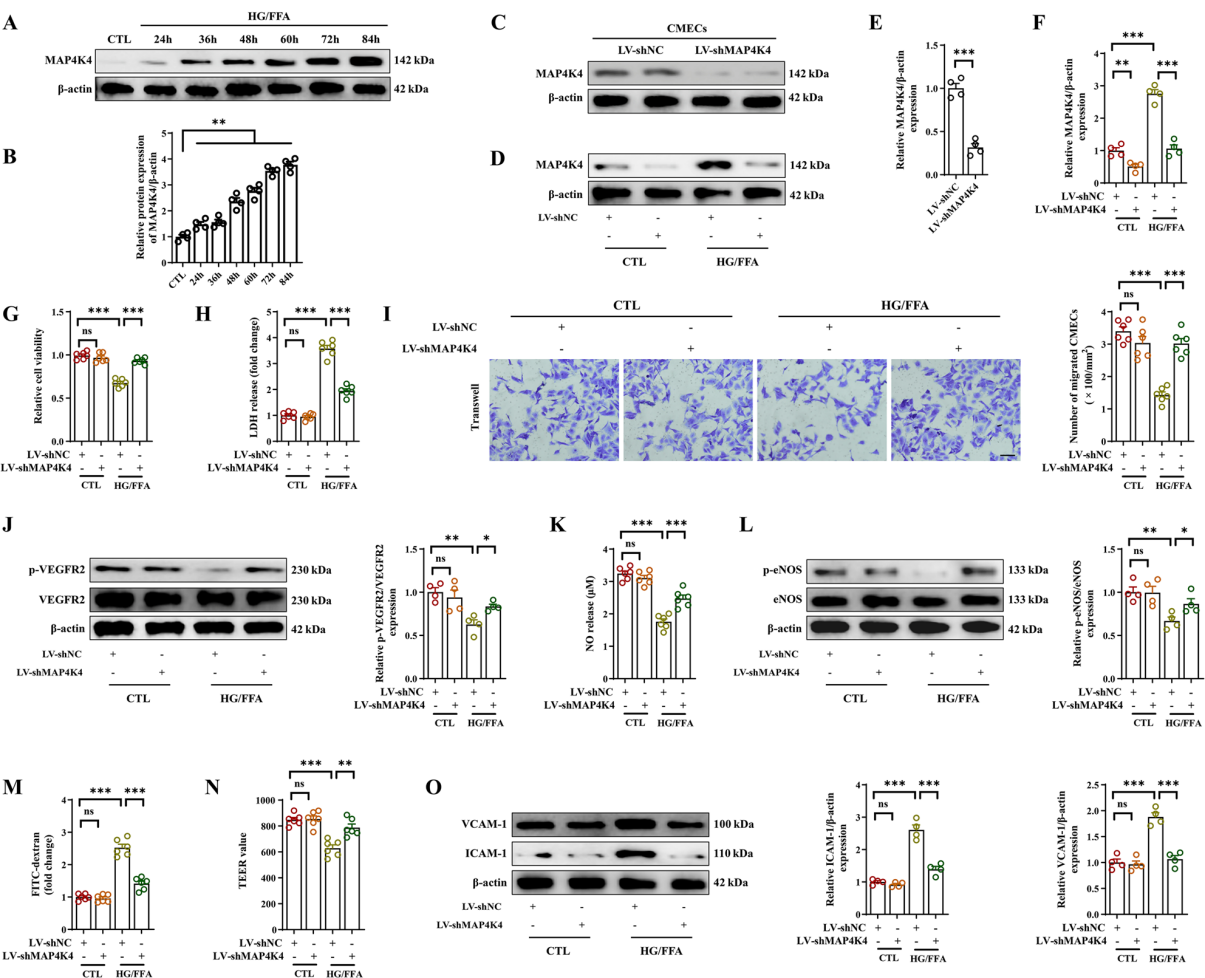
MAP4K4 knockdown ameliorated cardiac microvascular endothelial dysfunction under HG/FFA conditions. Cultured CMECs were transfected with LV-shMAP4K4 for 48 h and exposed to HG/FFA conditions. **A–B** Representative western blot and related statistical analysis of MAP4K4 protein expression in CMECs under HG/FFA conditions. **C–F** The transfection efficiency of LV-shMAP4K4 under control and HG/FFA conditions was measured via western blotting. **G** Relative cell viability was determined by a CCK-8 assay. **H** Statistical analysis of LDH release. **I** Representative images of the transwell assay and statistical analysis of migrated cells. Scale bars: 100 μm. **J** Total and phosphorylated VEGFR2 protein expression and quantitative analysis of VEGFR2. **K** Statistical analysis of NO release. **L** Total and phosphorylated protein expression and quantitative analysis of eNOS. **M** Statistical analysis of FITC-dextran permeability in the indicated groups. **N** TEER was analyzed to assess the barrier function of endothelial cells. **O** Protein expression and quantitative analysis of VCAM-1 and ICAM-1. *p < 0.05, **p < 0.01, ***p < 0.001 indicate significant differences. Four to six biological replicates were performed, and the results are indicated in scatter plots

### Silencing MAP4K4 improved endothelial function under HG/FFA conditions

To further evaluate MAP4K4 functions in vitro, HCMECs were used to assess the effects of MAP4K4 on endothelial dysfunction under HG/FFA conditions. The protein expression of MAP4K4 gradually increased under HG/FFA conditions over time (Fig. 2A, B). Thus, HCMECs were subjected to HG/FFA injury for 72 h to construct an in vitro endothelial injury model of diabetes. Additionally, MAP4K4 was knocked down with LV-shMAP4K4 to assess the effect of MAP4K4 on cell function (Fig. 2C–F). Cell viability was notably decreased, and LDH release was markedly increased by HG/FFA injury (Fig. 2G, H). These effects were significantly reversed by MAP4K4 knockdown, suggesting that silencing MAP4K4 led to a significant improvement in endothelial protection (Fig. 2G, H).

VEGFR2 activation promotes EC migration and proliferation. MAP4K4 knockdown significantly ameliorated the decreases in EC migration and VEGFR2 phosphorylation caused by HG/FFA (Fig. 2I, J). Subsequently, we assessed the effects of silencing MAP4K4 on endothelial-dependent vasodilation. After HG/FFA injury, the NO synthesis capacity and eNOS phosphorylation of HCMECs were greatly decreased, but these effects were markedly ameliorated via MAP4K4 knockdown (Fig. 2K, L). FITC-dextran permeation and TEER were measured to evaluate the effects of MAP4K4 on endothelial permeability. The results showed a significant increase in FITC-dextran permeation and a noticeable decrease in TEER following HG/FFA treatment (Fig. 2M, N). In contrast, MAP4K4 knockdown decreased FITC-dextran permeation and enhanced the TEER (Fig. 2M, N), indicating that suppression of MAP4K4 expression maintained endothelial integrity and improved barrier function. In addition, silencing MAP4K4 suppressed endothelial inflammation, as evidenced by inhibited VCAM-1 and ICAM-1 protein expression under HG/FFA injury (Fig. 2O). Collectively, the aforementioned data indicate a potential role for MAP4K4 in triggering CMEC dysfunction caused by HG/FFA.

### MAP4K4 knockdown improved mitochondrial homeostasis in CMECs after HG/FFA injury

**Fig. 3:**
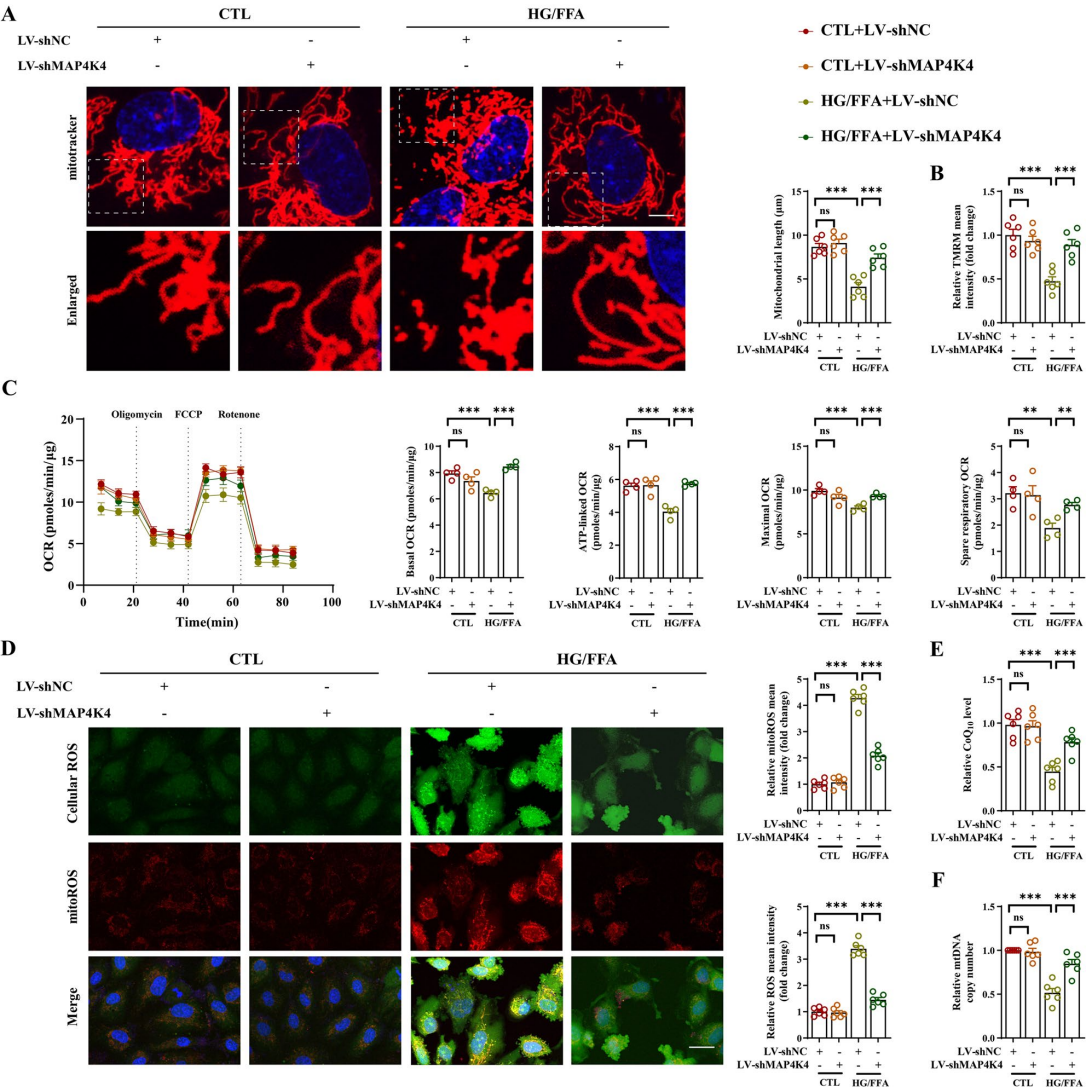
MAP4K4 knockdown improved mitochondrial morphology and function. HCMECs were transfected with LV-shMAP4K4 for 48 h and exposed to HG/FFA conditions for 72 h. **A** Mitochondrial morphology (red) was visualized by MitoTracker staining, and mitochondrial length was quantified. Scale bar = 10 μm. **B** Mitochondrial membrane potential was detected by TMRM staining. **C** Mitochondrial respiratory activity was assessed using a Seahorse analyzer. The baseline OCR, ATP-linked OCR, maximal OCR and spare respiratory OCR were quantitatively analyzed. **D** Intracellular ROS (green) and mitoROS (red) were photographed and quantitatively analyzed. Scale bar = 25 μm. **E** Quantitative analysis of the CoQ10 concentration in CMECs. **F** Quantification of the mtDNA copy number in each group. *p < 0.05, **p < 0.01, ***p < 0.001 indicate significant differences. Four to six biological replicates were performed, and the results are indicated in scatter plots

Since endothelial dysfunction is closely correlated with mitochondrial functional and structural damage during HG/FFA injury, we explored the effects of silencing MAP4K4 on mitochondrial function [2]. HG/FFA injury promoted mitochondrial network disruption and triggered mitochondrial fragmentation, whereas LV-shMAP4K4 transfection significantly inhibited mitochondrial fission and improved the mitochondrial network (Fig. 3A). In addition, we found that HG/FFA injury significantly reduced the MMP, as shown by reduced TMRM intensity, but this effect was completely abolished by MAP4K4 knockdown (Fig. 3B). Mitochondrial respiration is an important parameter of mitochondrial function [33]. After HG/FFA injury, the classic indices of mitochondrial respiration, including the basal OCR, ATP-linked OCR, maximal OCR, and spare respiratory OCR, were significantly reduced; however, MAP4K4 knockdown ameliorated mitochondrial respiratory dysfunction (Fig. 3C).

HG/FFA injury led to intracellular ROS and mitoROS accumulation, and these effects were alleviated by silencing MAP4K4 (Fig. 3D). CoQ10 is an important mitochondrion-targeted lipid-soluble antioxidant [36]. HG/FFA treatment reduced mitochondrial DNA copy number and CoQ10 levels (Fig. 3E, F). However, silencing MAP4K4 significantly improved the mitochondrial DNA copy number and CoQ10 levels after HG/FFA injury (Fig. 3E, F). Together, the present data strongly indicate that silencing MAP4K4 exerts protective effects on endothelial mitochondrial structure and function.

### Increased MAP4K4 expression enhanced SNO-Drp1 and promoted Drp1 translocation to mitochondria

**Fig. 4:**
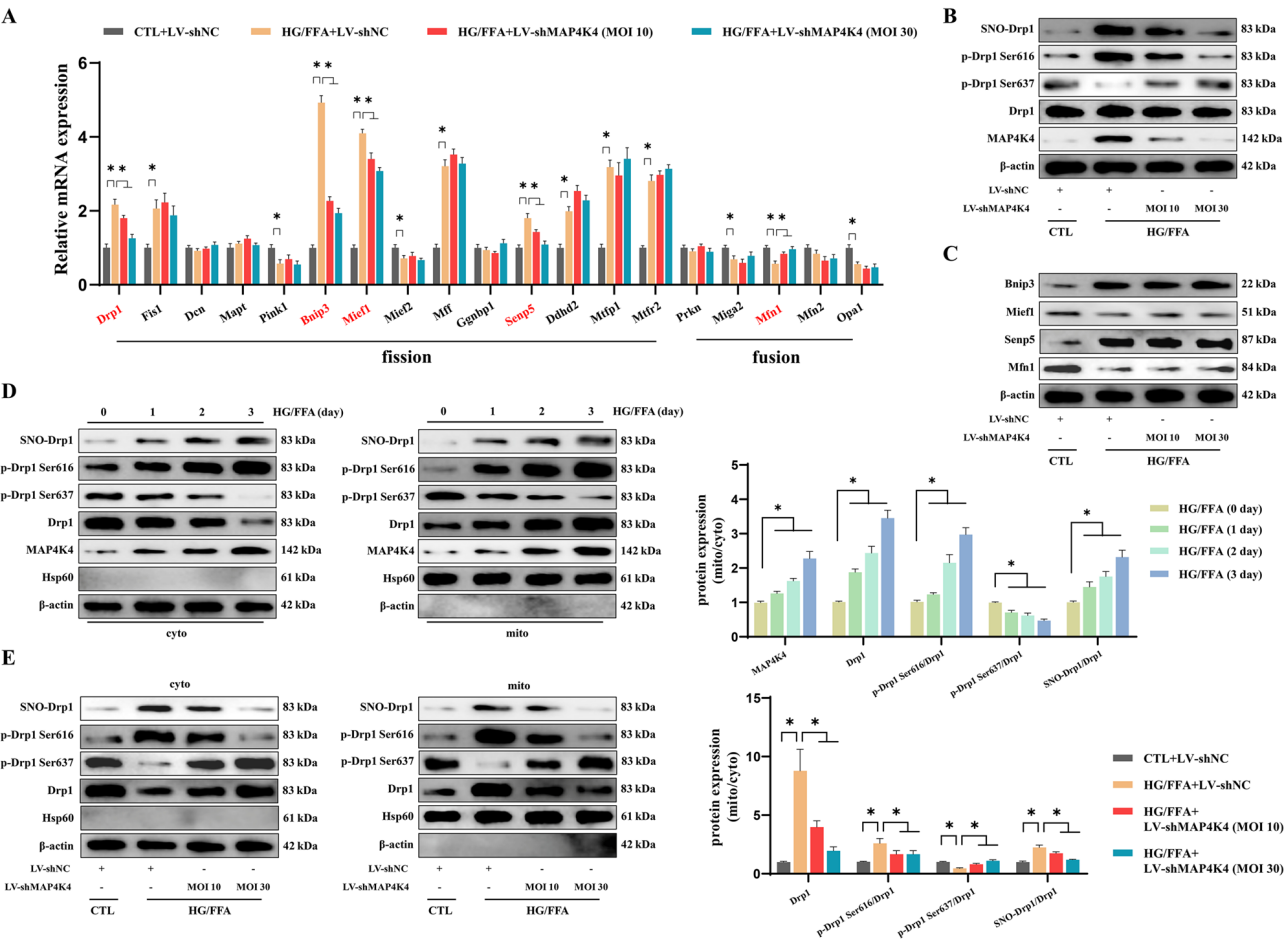
MAP4K4 silencing inhibited the mitochondrial translocation of Drp1 and downregulated SNO-Drp1. HCMECs were exposed to HG/FFA conditions for 72 h, with or without transfection with LV-shMAP4K4. **A** Relative mRNA expression of mitochondrial dynamics-related genes. **B** Representative immunoblotting images showing the protein expression, phosphorylation, and S-nitrosylation of Drp1. **C** Representative immunoblotting images of Bnip3, Mief1, Senp5, and Mfn1. **D** Mitochondrial and cytoplasmic expression of MAP4K4 and Drp1, Drp1 phosphorylation at Ser616, Drp1 phosphorylation at Ser637, and SNO-Drp1 were assessed by western blotting and statistically analyzed under HG/HFFA conditions. **E** Mitochondrial and cytoplasmic expression of Drp1, Drp1 phosphorylation at Ser616, Drp1 phosphorylation at Ser637, and SNO-Drp1 were assessed by western blotting and statistically analyzed after silencing MAP4K4. *p < 0.05, **p < 0.01, ***p < 0.001 indicate significant differences. Four biological replicates were performed, and the results are indicated in scatter plots

Based on the finding that silencing MAP4K4 ameliorated abnormal mitochondrial dynamics under HG/FFA injury, we further explored whether MAP4K4 could regulate mitochondrial fusion-/fission-related genes. The results indicated that among the 19 genes associated with mitochondrial dynamics, 14 exhibited altered expression after HG/FFA injury. Notably, the changes in the mRNA expression of Drp1, Bnip3, Mief1, Senp5 and Mfn1 were significantly reversed following LV-MAP4K4 transfection (Fig. 4A).

Given this finding, western blotting was used to investigate the protein expression of the above genes. MAP4K4 had no obvious effect on the protein level of Drp1 after HG/FFA injury. However, silencing MAP4K4 significantly inhibited the phosphorylation of Drp1 at serine 616 (Ser616) and promoted the phosphorylation of Drp1 at serine 637 (Ser637) under HG/FFA conditions (Fig. 4B). No significant correlations were found between MAP4K4 and the other proteins (Fig. 4C). It has been previously shown that S-nitrosylation can lead to Drp1 activation and excessive mitochondrial fission [24, 37]. Similarly, we found that SNO-Drp1 levels were significantly increased after HG/FFA injury, while this effect was strongly decreased by MAP4K4 knockdown (Fig. 4B).

Drp1 translocates to mitochondria via its ligands and thereby promotes mitochondrial fission [20]. In the present study, the mitochondrial translocation of Drp1 gradually increased in a time-dependent manner under HG/FFA conditions (Fig. 4D). This process was accompanied by increased phosphorylation of Drp1 at Ser616 and SNO-Drp1 (Fig. 4D). In contrast, the phosphorylation of Drp1 at Ser637 in mitochondria gradually decreased with prolonged injury time (Fig. 4D). Interestingly, MAP4K4 also translocated to mitochondria under conditions of HG/FFA injury (Fig. 4D). In contrast, MAP4K4 knockdown markedly inhibited the HG/FFA-induced translocation of Drp1 to mitochondria (Fig. 4E). Moreover, silencing MAP4K4 inhibited SNO-Drp1, reduced Drp1 Ser616 phosphorylation, and promoted Drp1 Ser637 phosphorylation under HG/FFA conditions in both the cytosol and mitochondria (Fig. 4E). These results suggested that MAP4K4 promoted Drp1 activation and in turn aggravated mitochondrial fission under HG/FFA conditions (Fig. 4D, E).

Taken together, these results strongly suggest that MAP4K4 negatively regulates mitochondrial dynamics by promoting SNO-Drp1 and driving the mitochondrial translocation of Drp1.

### Knockdown of MAP4K4 decreased SNO-Drp1 levels and inhibited Drp1 translocation to mitochondria by upregulating the expression of GPX4

**Fig. 5:**
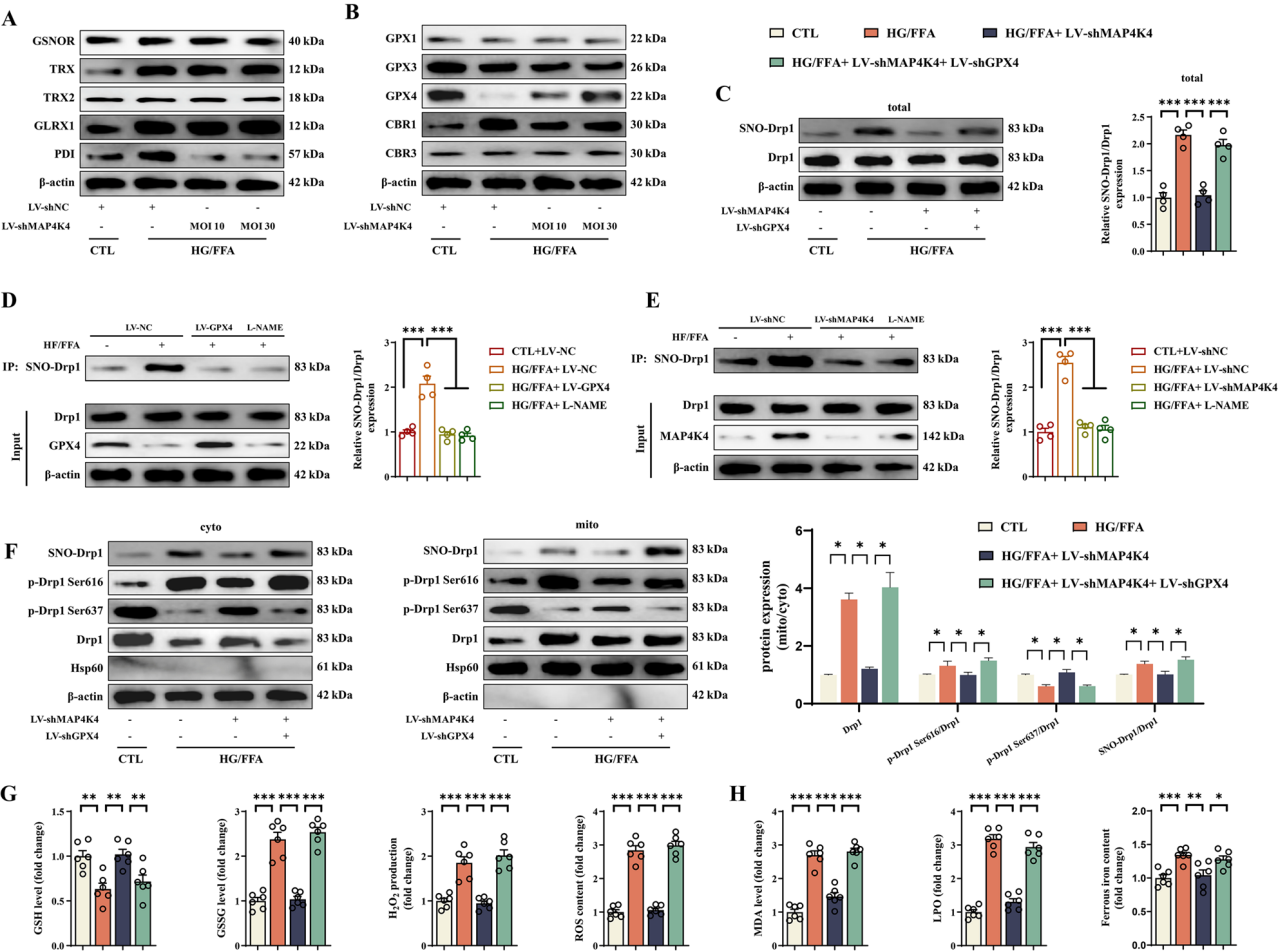
MAP4K4 modulated the SNO-Drp1 through GPX4. **A–B** HCMECs were transfected with LV-shMAP4K4 for 48 h and exposed to HG/FFA conditions for 72 h. Representative western blot and statistical analysis of S-nitrosylation modification-related proteins. **C** HCMECs were cotransfected with LV-shMAP4K4 and LV-shGPX4 and subjected to HG/FFA injury. SNO-Drp1 was assessed by western blotting. **D** HCMECs were transfected with LV-GPX4 or treated with L-NAME and then subjected to HG/FFA injury. SNO-Drp1 was assessed by western blotting. **E** HCMECs were treated with LV-shMAP4K4 or L-NAME and subjected to HG/FFA injury. SNO-Drp1 expression was then assessed by western blotting. **F** The mitochondrial and cytoplasmic expression of Drp1 and the S-nitrosylation and phosphorylation of Drp1 were assessed by western blotting. **G** Quantitative analysis of GSH, GSSG, H_2_O_2_ and ROS levels in the indicated groups. **H** Quantitative analysis of the MDA, LPO, and iron content in the indicated groups. *p < 0.05, **p < 0.01, ***p < 0.001 indicate significant differences. Four biological replicates were performed, and the results are indicated in scatter plots

Further work was carried out to test the signaling pathways involved in the regulation of S-nitrosylation [38, 39]. The protein expression of TRX, GLRX1, PDI and CBR1 increased under HG/FFA injury, whereas that of GPX3 and GPX4 decreased (Fig. 5A, B). Among these genes, only PDI, GPX4 and CBR1 exhibited opposite expression patterns upon silencing MAP4K4 (Fig. 5A, B). However, only knockdown of GPX4 abolished the effect of MAP4K4 silencing on SNO-Drp1 (Fig. 5C, Additional file 2: Fig. S2A). Neither PDI nor CBR1 overexpression had such an effect (Additional file 2: Fig. S2B–E). In addition, a co-IP assay revealed a direct interaction between MAP4K4 and GPX4 (Additional file 2: Fig. S2F). These results suggest that MAP4K4 knockdown may downregulate SNO-Drp1 by directly targeting GPX4. To further confirm the role of GPX4 in SNO-Drp1, GPX4 was overexpressed in HCMECs (Fig. S2G). The overexpression of GPX4 obviously inhibited SNO-Drp1, similar to the effects of MAP4K4 knockdown and treatment with L-NAME, an SNO-Drp1 inhibitor (Fig. 5D, E) [29]. As previously demonstrated, transfection of HCMECs with LV-shMAP4K4 under HG/FFA injury obviously inhibited the mitochondrial translocation of Drp1, and this effect was accompanied by suppression of mitochondrial SNO-Drp1, a reduction in Drp1 phosphorylation at Ser616, and an increase in Drp1 phosphorylation at Ser637 (Fig. 5F). However, these protective effects were counteracted by cotransfection with LV-shGPX4 (Fig. 5F).

Considering that GPX4 has been widely used to alleviate oxidative stress and ferroptosis, the present study further investigated whether MAP4K4 could stimulate oxidative stress and ferroptosis [40]. After HG/FFA injury, the GSH content was obviously reduced, while the GSSG content, H_2_O_2_ production and ROS content were increased (Fig. 5G). The above oxidative stress injuries were obviously alleviated by MAP4K4 knockdown (Fig. 5G). Moreover, the MDA level, lipid peroxidation and ferrous iron content increased after HG/FFA injury but were largely attenuated by MAP4K4 knockdown (Fig. 5H). However, the protective effects of MAP4K4 silencing on oxidative stress and ferroptosis were negated by the downregulation of GPX4 (Fig. 5G, H). Moreover, GPX4 downregulation abolished the effects of MAP4K4 silencing on cell viability and LDH release (Fig. S2H). These results confirmed that MAP4K4 could enhance SNO-Drp1 and in turn induce mitochondrial and cellular dysfunction by downregulating GPX4.

### MAP4K4 promoted Drp1 S-nitrosylation at C644 and in turn led to CMEC dysfunction under HG/FFA conditions

**Fig. 6:**
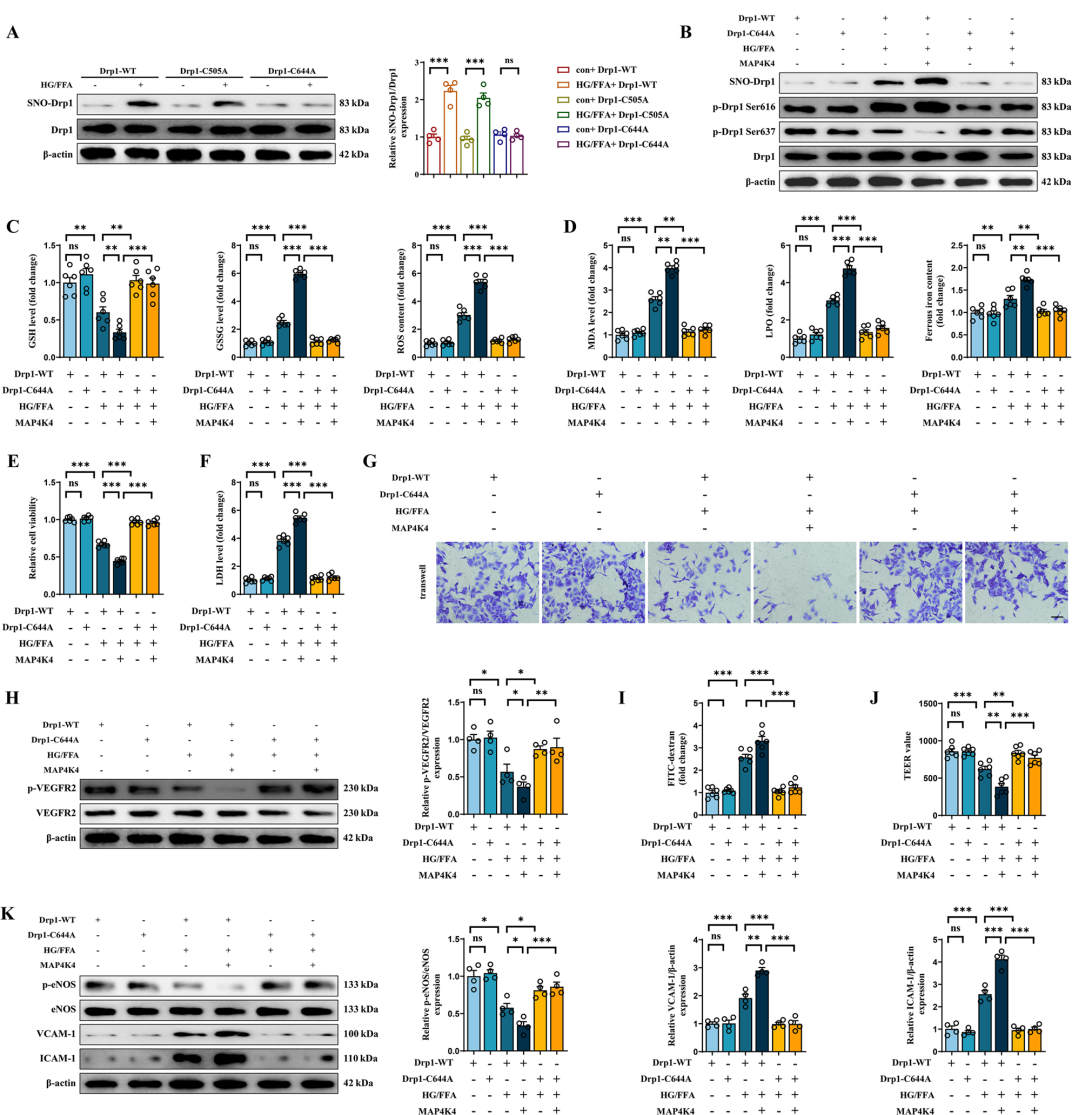
The C644A mutation inhibited SNO-Drp1 and reversed the effects of MAP4K4 on CMEC dysfunction under HG/FFA conditions. **A** Relative SNO-Drp1 levels were assessed in the Drp1-WT cell line, the Drp1-C505A knock-in cell line and the Drp1-C644A knock-in cell line, with or without HG/FFA injury.** B** MAP4K4 was overexpressed in Drp1-WT and Drp1-C644A knock-in cells treated with HG/FFA. The protein expression, S-nitrosylation and phosphorylation of Drp1 were assessed by western blotting. **C** Quantitative analysis of GSH, GSSG, and ROS levels in the indicated groups. **D** Quantitative analysis of the MDA, LPO, and ferrous iron content in the indicated groups. **E–F** Relative cell viability was determined by the CCK-8 assay, and cytotoxicity was measured by the LDH release assay.** G** Representative images of the transwell assay. Scale bars: 100 μm. **H** The expression and phosphorylation of VEGFR2 were assessed by western blotting. **I** Statistical analysis of FITC-dextran permeability.** J** TEER was used to assess the barrier function of endothelial cells. **K** Protein expression and quantitative analysis of p-eNOS, eNOS, VCAM-1 and ICAM-1. *p < 0.05, **p < 0.01, ***p < 0.001 indicate significant differences. Four to six biological replicates were performed, and the results are indicated in scatter plots

Next, we examined the potential cysteine residues of Drp-1 involved in S-nitrosylation in diabetes. According to previous studies [23, 41], C505 and C644 were identified as possible residues of Drp1 in humans. In addition, C505 and C644 are highly conserved across species (Fig. S3A, B). Therefore, the Drp1-C505A and Drp1-C644A knock-in cell lines were constructed with CRISPR‒Cas9 (Fig. S3C, D). The results suggested that only the C644A mutation attenuated SNO-Drp1 formation under HG/FFA conditions (Fig. 6A). Moreover, even though MAP4K4 overexpression enhanced SNO-Drp1 levels and promoted Ser616 phosphorylation of Drp1 in wild-type HCMECs, it did not change the S-nitrosylation or phosphorylation of Drp1 in the Drp1-C644A cell line. (Fig. 6B, S3E). The above results suggested that C644 was the cysteine residue for SNO-Drp1 in diabetes and acted as a potential target for MAP4K4.

The present work also demonstrated that MAP4K4 overexpression accentuated oxidative stress, ferroptosis and cell cytotoxicity in the wild-type cell line but had no effect on the Drp1-C644A cell line under HG/FFA conditions (Fig. 6C–F, Fig. S3F). In addition, the Drp1-C644A mutation itself rendered endothelial cells more resistant to HG/FFA injury (Figs. 6C–F, Additional file 3: Fig. S3F). Attempts were then made to assess the role of C644 of Drp1 in endothelial functions. The C644A mutation significantly increased VEGFR2 phosphorylation and cell migration under HG/FFA injury and abolished the inhibitory effects of MAP4K4 on VEGFR2 phosphorylation and cell migration (Fig. 6G, H, Additional file 3: Fig. S3G). In addition, MAP4K4 promoted endothelial barrier collapse, the effects of which were negated by the C644A mutation (Fig. 6I, J). Furthermore, the Drp1-C644A mutation improved endothelium-dependent vasodilatation and inflammatory responses, as evidenced by increased eNOS phosphorylation and NO content and suppressed VCAM-1 and ICAM-1 expression (Fig. 6K, Additional file 3: Fig. S3H).

### Mouse C650A mutation abolished Drp1-induced cardiac microvascular dysfunction in diabetes

C650 of mouse Drp1 is identical to C644 of human Drp1 (Additional file 3: Fig. S3B). To further define this functional target site of Drp1, we constructed an AAV9 to achieve endothelial-specific overexpression of Drp1-WT or Drp1-C650A in db/m and db/db mice (Additional file 4: Fig. S4A, B).

**Fig. 7:**
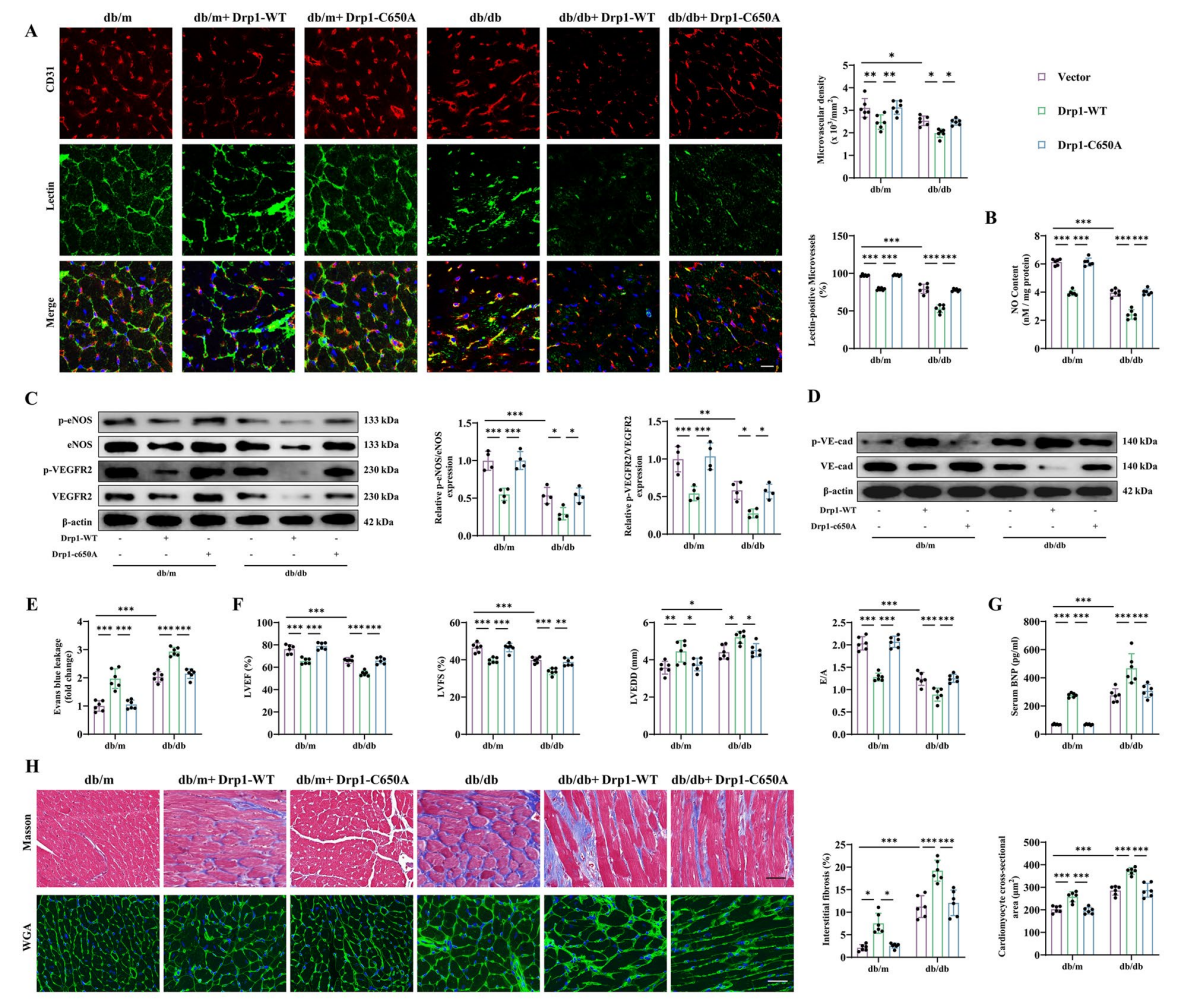
C650 mutation inhibited Drp1 SNO, attenuated cardiac microcirculatory injury and ameliorated ventricular remodeling in db/db mice. Four-week-old male db/db mice and age-matched db/m mice were transfected with AAV9-Drp1-WT or AAV9-Drp1-C650A for 24 weeks. **A** Cardiac microvascular density was detected by immunofluorescence staining of CD31, and microvascular blood flow was assessed by a lectin-FITC perfusion assay. Scale bar = 25 μm. **B** Quantitative analysis of NO content in myocardial tissues. **C** Total and phosphorylated protein expression of eNOS and VEGFR2. **D** Total and phosphorylated VE-cadherin protein levels. **E** Quantitative analysis of EB leakage in each group. **F** Statistical analysis of the LVEF, LVFS, LVEDD and E/A ratio in the indicated groups. **G** Quantitative analysis of serum BNP levels. **H** Cardiac fibrosis and the cardiomyocyte cross-sectional area were detected by Masson trichrome staining and WGA staining, respectively. Scale bar = 70 μm. *p < 0.05, **p < 0.01, ***p < 0.001 indicate significant differences. Four to six biological replicates were performed, and the results are indicated in scatter plots

Overexpression of Drp1-WT compromised endothelial-dependent vasodilatation and angiogenesis, as evidenced by reduced cardiac microvascular reperfusion, suppressed eNOS and VEGFR2 phosphorylation, and reduced NO content (Fig. 7A–C). In contrast, C650A-mutant Drp1 did not reduce vasodilatation or angiogenesis in either db/m or db/db mice (Fig. 7A–C). As we have demonstrated before, diabetes promoted microvascular leakage via VE-cadherin phosphorylation, the effects of which were aggravated by Drp1-WT overexpression but were not intensified by Drp1-C650A (Fig. 7D, E). Moreover, the C650A mutation abolished the promotive effects of Drp1 on VCAM-1 and ICAM-1 expression in diabetic hearts (Fig. S4C). The results also showed that the C650A mutation significantly weakened the stimulatory effects of Drp1 on oxidative stress and ferroptosis (Additional file 4: Fig. S4D, E).

More importantly, Drp1-WT overexpression exacerbated cardiac systolic and diastolic dysfunction and promoted cardiac hypertrophy and interstitial fibrosis in db/db mice (Fig. 7F–H). In contrast, the above effects were not observed after overexpression of the C650A mutant Drp1 (Fig. 7F–H). These results further confirmed that the negative effects of Drp1 on cardiac microvascular injury and cardiac dysfunction in diabetes are primarily dependent on S-nitrosylation at C650 in mice.

### MAP4K4-inducible Drp1 S-nitrosylation promoted ferroptosis by activating Drp1 in db/db mice

To further validate the regulatory effects of MAP4K4 on SNO-Drp1, DMX-5804, a specific MAP4K4 inhibitor, was used [28]. DMX-5804 decreased MAP4K4 expression in HG/FFA-injured cells and db/db mice (Additional file 5: Fig. S5A). As expected, DMX-5804 significantly reduced SNO-Drp1, decreased Drp1 phosphorylation at Ser616, increased Drp1 phosphorylation at Ser637, and inhibited Drp1 translocation to mitochondria after HG/FFA treatment (Additional file 5: Fig. S5B–D). In addition, DMX-5804 ameliorated the oxidative stress and ferroptosis caused by HG/FFA treatment (Additional file 5: Fig. S5E).

**Fig. 8:**
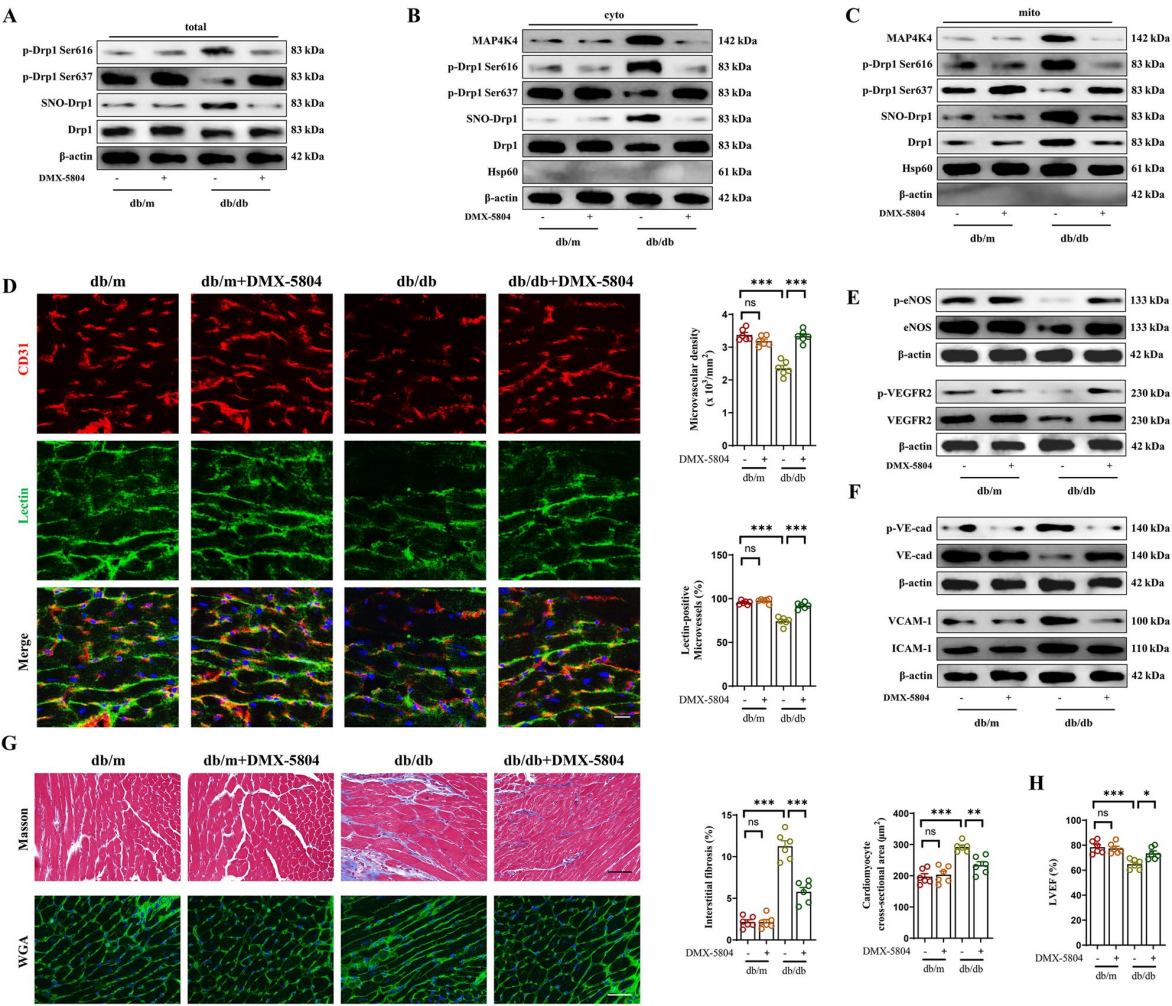
MAP4K4 contributed to myocardial ferroptosis by facilitating Drp1 S-nitrosylation and phosphorylation-induced activation in vivo. Four-week-old male db/db mice received 3 mg/kg DMX-5804 orally three times per week for 24 weeks. **A-C** Total, mitochondrial, and cytoplasmic levels of MAP4K4, Drp1, Drp1 phosphorylated at Ser616, Drp1 phosphorylated at Ser637, and S-nitrosylated Drp1 were assessed by western blotting. **D** Cardiac microvascular density was detected by immunofluorescence staining of CD31, and microvascular blood flow was assessed by a lectin-FITC perfusion assay. Scale bar = 25 mm. **E** Total and phosphorylated protein expression of eNOS and VEGFR2. **F** Total and phosphorylated VE-cadherin protein expression and the protein expression of VCAM-1 and ICAM-1. **G** Cardiac fibrosis and cardiomyocyte cross-sectional area were detected by Masson trichrome staining and WGA staining, respectively. Scale bar = 70 mm. **H** Statistical analysis of the LVEF. *p < 0.05, **p < 0.01, ***p < 0.001 indicate significant differences. Four to six biological replicates were performed, and the results are indicated in scatter plots

Similar to the in vitro results, DMX-5804 significantly abrogated SNO-Drp1, suppressed Drp1 phosphorylation at Ser616, and inhibited the mitochondrial translocation of Drp1 in db/db mice (Fig. 8A–C). In addition, DMX-5804 prevented oxidative stress and ferroptosis in the hearts of db/db mice (Additional file 5: Fig. S5F). In addition, DMX-5804 improved endothelial-dependent microvascular perfusion, stimulated angiogenesis, improved endothelial barrier function, and suppressed endothelial-associated inflammation in db/db mice (Fig. 8D–F). Moreover, DMX-5804 significantly improved cardiac function and ameliorated ventricular remodeling in db/db mice (Fig. 8G, H, Additional file 5: Fig. S5G, H). These data strongly support that MAP4K4 is a critical regulator of SNO-Drp1 and that inhibiting MAP4K4 can suppress oxidative stress and ferroptosis, thus inhibiting the development of microcirculatory disturbance in DCM.

## Discussion

Our study revealed that MAP4K4 regulates SNO-Drp1 by interfering with GPX4 in endothelial cells. In addition, human C644 (mouse C650) was identified as the core site of SNO-Drp1 in diabetic injury. Knockdown of MAP4K4 or treatment with a MAP4K4 inhibitor (DMX-5804) inhibited SNO-Drp1 and protected endothelial cells and cardiac microcirculation against diabetes by enhancing mitochondrial functions and suppressing oxidative stress injury and ferroptosis. Similarly, the human C644A (mouse C650A) mutation negated SNO-Drp1 and abolished the adverse effects of Drp1 in promoting endothelial ferroptosis and cardiac microvascular dysfunction. These data reveal that MAP4K4 and SNO-Drp1 promote the development of cardiac microvascular dysfunction in diabetes and provide theoretical evidence that the MAP4K4-GPX4-Drp1 signaling pathway is a novel therapeutic target for DCM.

MAP4K4 is a member of the Ste20 family of kinases, which have been broadly reported to participate in multiple cardiovascular diseases [9, 42]. MAP4K4 is activated in failing human hearts and induces oxidative stress to promote cell death in myocardial infarction models [43]. In contrast, MAP4K4 inhibition rescues mitochondrial function in cardiomyocytes, ameliorates apoptosis and reduces infarction size [43–45]. Pathological cardiac hypertrophy or amyotrophy precedes heart failure [8, 31]. Silencing of MAP4K4 inhibits the prohypertrophic factor NFAT in angiotensin II-treated hearts, and the inhibition of MAP4K4 preserves cardiomyocyte function after doxorubicin treatment [8, 46]. In addition to its role in cardiomyocytes, MAP4K4 is highly expressed in endothelial cells and participates in the inflammatory response [9]. EC-specific MAP4K4 deletion alleviates aortic atherosclerosis by decreasing macrophage permeation and lipid accumulation [13, 47]. More importantly, one study demonstrated that animals lacking endothelial MAP4K4 were protected from skeletal muscle microvascular rarefaction via the suppression of endothelial senescence and increased metabolic capacity in obese mice [48]. However, the role and intrinsic mechanism of MAP4K4 in diabetes-induced microvascular dysfunction have not been explored. The present study demonstrated that silencing or inhibiting MAP4K4 (DMX-5804) in DCM significantly improved microvascular density, angiogenesis, and endothelial-dependent microvascular perfusion by activating the VEGF and eNOS signaling pathways. Chronic myocardial interstitial edema and the inflammatory response contribute to myocardial fibrosis, cardiac remodeling, and heart dysfunction [2, 6, 49]. Inhibiting MAP4K4 significantly improved endothelial barrier function and endothelium-related inflammatory responses, ultimately improving cardiac remodeling and dysfunction. These data imply that cardiac or EC-specific MAP4K4 inhibition confers cardiovascular protection against long-term diabetes.

The roles of Drp1 in mitochondrial homeostasis, myocardial microcirculatory disturbance and DCM have been widely investigated, with a major focus on the phosphorylation of Drp1 at Ser616 and Ser637 [50, 51]. In addition to being phosphorylated, Drp1 can undergo multiple posttranslational modifications, such as palmitoylation, SUMOylation, acetylation, and ubiquitination [21, 52]. Importantly, accumulating evidence has demonstrated the involvement of SNO-Drp1 in facilitating mitochondrial fission, promoting mitochondrial dysfunction, and triggering mitochondria-dependent cell death [29, 53]. In addition, S-nitrosylation disturbs the phosphorylation of Drp1 between Ser616 and Ser637 [29]. Nine cysteine residues of Drp1 are potential S-nitrosylation sites, among which human C505 and C644 have been verified [23]. The mouse C650A (human C644A) mutation inhibits SNO-Drp1 in isoprenaline-induced heart failure[24]. However, little is known about the role of SNO-Drp1 in diabetes and endothelial injury. Here, our findings demonstrated that diabetes promoted SNO-Drp1, and human C644, rather than C505, was identified as the cysteine site of Drp1 for S-nitrosylation. Additionally, S-nitrosylation of Drp1 at C644 was revealed as a downstream signaling pathway for MAP4K4 in diabetes and may be a potential target for cardiovascular protection.

As a classic GSH peroxidase-related protein, GPX4 has been suggested to participate in a variety of biological functions, such as the regulation of oxidative stress and cell death, via its intrinsic role in modulating the GSH/GSSG balance [54, 55]. In addition, GSH levels are closely related to S-nitrosylation in a variety of proteins [56, 57]. The present data revealed that MAP4K4 could regulate multiple proteins that modulate S-nitrosylation, among which only GPX4 inhibited SNO-Drp1 in diabetic injury. GPX4 is widely known to inhibit ferroptosis [58]. Interestingly, Drp1 has recently been reported to modulate the occurrence and development of glioma ferroptosis [59]. Additionally, Drp1 oligomerization can promote ferroptosis, which in turn suppresses hepatocellular carcinoma cell growth [60]. However, it is not clear whether SNO-Drp1 can promote ferroptosis in endothelial injury in diabetes. The present study demonstrated that diabetes promoted SNO-Drp1 and promoted ferroptotic injury in endothelial cells. In contrast, MAP4K4 silencing, DMX-5804 treatment or the human Drp1 C644A mutation inhibited SNO-Drp1 and alleviated endothelial ferroptotic injury in diabetes, thereby improving cardiac microvascular disorders. Therefore, in addition to affecting apoptosis, the present study identified MAP4K4 as a crucial regulator of ferroptosis in diabetes, and this process involves the GPX4-dependent GSH/GSSG balance and the S-nitrosylation of Drp1.

Even though the present study highlights novel findings that explain the potential of the MAP4K4-GPX4-Drp1 pathway in facilitating cardiac microvascular disorder in diabetes, several potential biases and limitations should be considered. The present work used db/db mice to verify our assumptions, which were not reconfirmed by other preclinical models of metabolic and cardiovascular diseases, such as ob/ob, Apoe^−/−^ or Ldlr^−/−^ mouse models. Previous reports identified MAP4K4 as an upstream signal for the AMPK-mTOR pathway, Hippo pathway, and JNK pathway [9]. However, the mechanisms by which diabetes stimulates MAP4K4 expression have not yet been investigated. The present work suggested that MAP4K4 modulates S-nitrosylation and endothelial ferroptosis by balancing GSH/GSSH from GPX4; however, the intermediary steps were not identified, and other complex signaling networks were not ruled out. Similarly, the source of NO and the direct effects of MAP4K4, which participates in the S-nitrosylation of Drp1, were not confirmed, considering eNOS and NO are reduced by diabetes. Studies aimed at solving the above issues in DCM are warranted.

To summarize, our study clearly presents evidence that MAP4K4 promotes SNO-Drp1 by suppressing the expression of GPX4, which induces endothelial ferroptosis and dysfunction and ultimately leads to cardiac microvascular disorders in DCM. This research therefore underscores novel potential roles of MAP4K4 and SNO-Drp1 in DCM and suggests that MAP4K4 or SNO-Drp1 may be novel therapeutic targets for endothelial ferroptosis.

### Supplementary Information

**Additional file 1: Figure S1**. Four-week-old male db/db mice and age-matched db/m mice were transfected with AAV9-shMAP4K4 or AAV9-shNC for 24 weeks. (A): Immunofluorescence staining of Flag-tagged AAV9 (green) and CD31-labeled CMECs (red) and statistical graphs of the percentage of Flag-positive CMECs. Scale bar = 50 mm. (B): The transfection efficiency of AAV9-shMAP4K4 in cardiac tissue was measured by western blotting. (C): Statistical analysis of the E/A ratio. *p < 0.05, **p < 0.01, ***p < 0.001 indicate significant differences. Four to six biological replicates were performed, and the results are indicated in scatter plots.

** Additional file 2: Figure S2**. (A): The transfection efficiency of LV-shGPX4 was measured via western blotting. (B): The transfection efficiency of LV-PDI was measured via western blotting. (C): HCMECs were cotransfected with LV-shMAP4K4 and LV-PDI and subjected to HG/FFA injury. SNO-Drp1 was assessed by western blotting. (D): The transfection efficiency of LV-CBR1 was measured via western blotting. (E): HCMECs were cotransfected with LV-shMAP4K4 and LV-CBR1 and subjected to HG/FFA injury. SNO-Drp1 was assessed by western blotting. (F): A co-IP assay was carried out using an antibody against GPX4, and western blotting was performed for MAP4K4 and GPX4. (G): The transfection efficiency of LV-GPX4 was measured via western blotting. (H): HCMECs were cotransfected with LV-shMAP4K4 and LV-shGPX4 and subjected to HG/FFA injury. Relative cell viability was determined by a CCK-8 assay, and cytotoxicity was measured by an LDH release assay. *p < 0.05, **p < 0.01, ***p < 0.001 indicate significant differences. Four to six biological replicates were performed, and the results are indicated in scatter plots.

** Additional file 3: Figure S3**. (A): Comparison of the sequence similarity of Drp1 across different species. Homo sapiens Drp1 497–514, Mus musculus Drp1 503–520, Rattus norvegicus Drp1 510–527, Bos taurus Drp1 510–527, and Danio rerio Drp1 496–513. The Drp1 C505 site in humans is highly conserved across different species. (B): Comparison of the sequence similarity of Drp1 across different species. Homo sapiens Drp1 636–653, Mus musculus Drp1 642–659, Rattus norvegicus Drp1 655–672, Bos taurus Drp1 649–666, and Danio rerio Drp1 591–608. The Drp1 C644 site in humans and the C650 site in mice are highly conserved across different species. (C): Genomic DNA was extracted from the indicated cells with or without C505A knockdown. PCR products were amplified and sequenced. (D): Genomic DNA was extracted from the indicated cells with or without C644A knockdown. PCR products were amplified and sequenced. (E): The transfection efficiency of LV-MAP4K4 was measured via western blotting. (F): Quantitative analysis of H_2_O_2_ content in the indicated groups. (G): Statistical analysis of the number of migrated cells in the Transwell assay. (H): Statistical analysis of NO release. *p < 0.05, **p < 0.01, ***p < 0.001 indicate significant differences. Four to six biological replicates were performed, and the results are indicated in scatter plots.

** Additional file 4: Figure S4**. (A): Immunofluorescence staining of His-tagged AAV9 (green) and CD31-labeled CMECs (red) and statistical graphs of the percentage of His-positive CMECs. Scale bar = 50 mm. (B): The transfection efficiency of AAV9-Drp1-WT and AAV9-Drp1-C650A in primary CMECs was measured by western blotting. (C): Protein expression of VCAM-1 and ICAM-1. (D): Quantitative analysis of GSH, GSSG, and ROS levels in the indicated groups. (E): Quantitative analysis of the MDA and LPO levels in the indicated groups. *p < 0.05, **p < 0.01, ***p < 0.001 indicate significant differences. Four to six biological replicates were performed, and the results are indicated in scatter plots.

** Additional file 5: Figure S5**. (A): Cells and db/db mice were treated with different concentrations of DMX-5804. The expression of MAP4K4 was detected by western blotting. (B): Representative immunoblotting images showing the protein expression, phosphorylation, and S-nitrosylation of Drp1 in HCMECs. (C-D): Mitochondrial and cytoplasmic levels of Drp1, Drp1 phosphorylated at Ser616, Drp1 phosphorylated at Ser637, and SNO-Drp1 were assessed by western blotting in HCMECs. (E): Quantitative analysis of ROS, MDA, LPO and ferrous iron content in HCMECs subjected to HG/FFA injury. (F): Quantitative analysis of ROS and LPO levels in cardiac tissues. (G): Statistical analysis of the LVFS, LVEDD, and E/A ratio data. (H): Quantitative analysis of serum BNP levels. *p < 0.05, **p < 0.01, ***p < 0.001 indicate significant differences. Four to six biological replicates were performed, and the results are indicated in scatter plots.

**Additional file 6: Table S1** Primer. **Table S2** Primary antibodies used in western blots.

## Data Availability

The datasets generated or analyzed in this study can be obtained upon reasonable request from the corresponding authors.
