# Supplementary figures and images for: MAP4K4 exacerbates cardiac microvascular injury in diabetes by facilitating S-nitrosylation modification of Drp1
Source: Cardiovasc Diabetol. 2024 May 9;23:164. doi: 10.1186/s12933-024-02254-7 (PMC11084109; doi:10.1186/s12933-024-02254-7)

**A**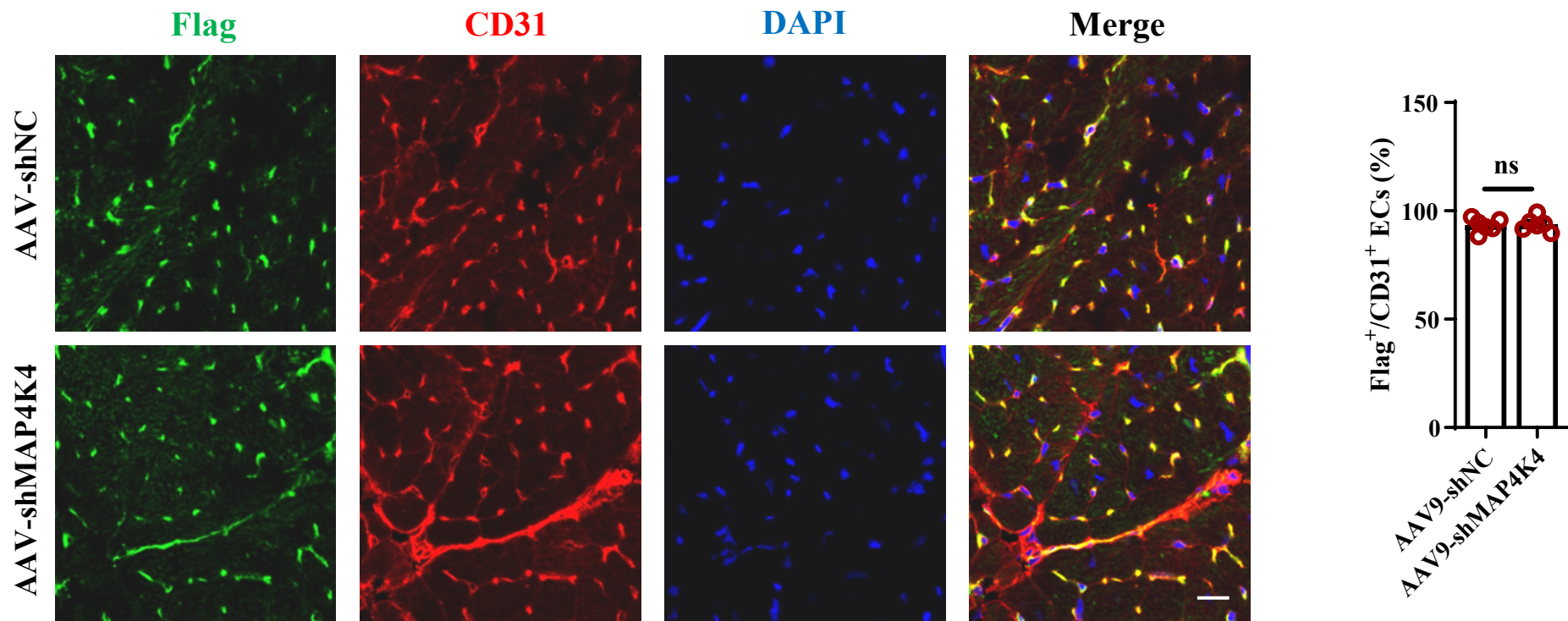**B**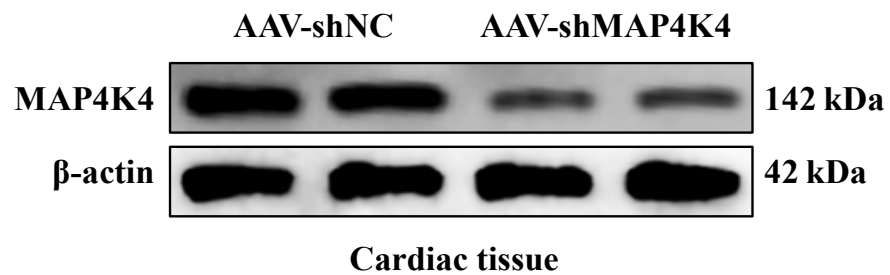**C**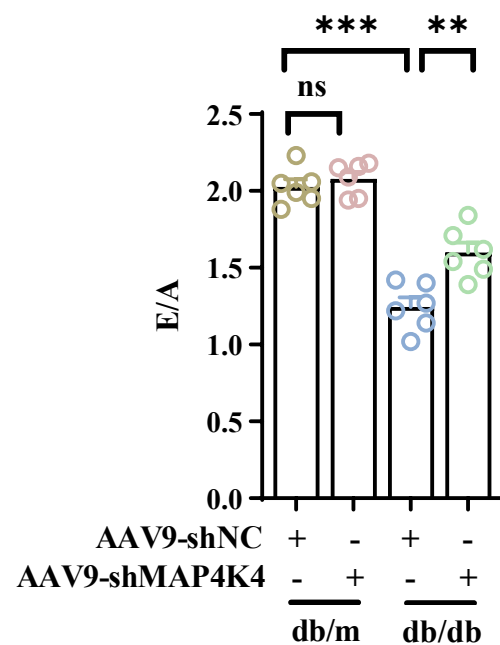

Supplement: Supplementary file 1 — Additional file 1: Figure S1. Four-week-old male db/db mice and age-matched db/m mice were transfected with AAV9-shMAP4K4 or AAV9-shNC for 24 weeks. (A): Immunofluorescence staining of Flag-tagged AAV9 (green) and CD31-labeled CMECs (red) and statistical graphs of the percentage of Flag-positive CMECs. Scale bar = 50 mm. (B): The transfection efficiency of AAV9-shMAP4K4 in cardiac tissue was measured by western blotting. (C): Statistical analysis of the E/A ratio. *p < 0.05, **p < 0.01, ***p < 0.001 indicate significant differences. Four to six biological replicates were performed, and the results are indicated in scatter plots. [file 12933_2024_2254_MOESM1_ESM.pdf]

**A**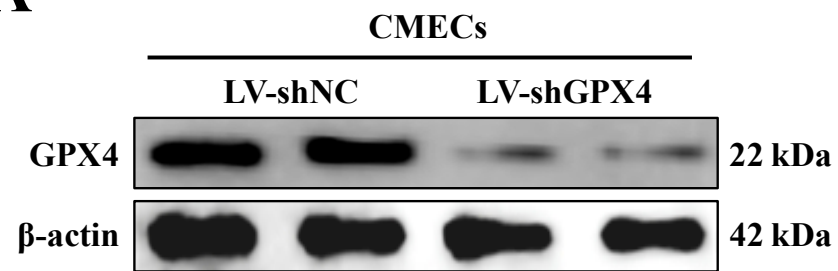**B**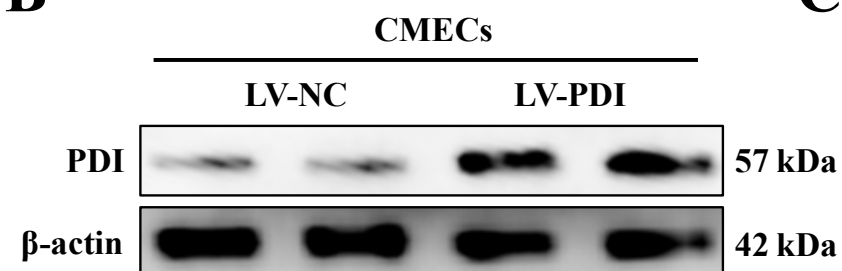**C**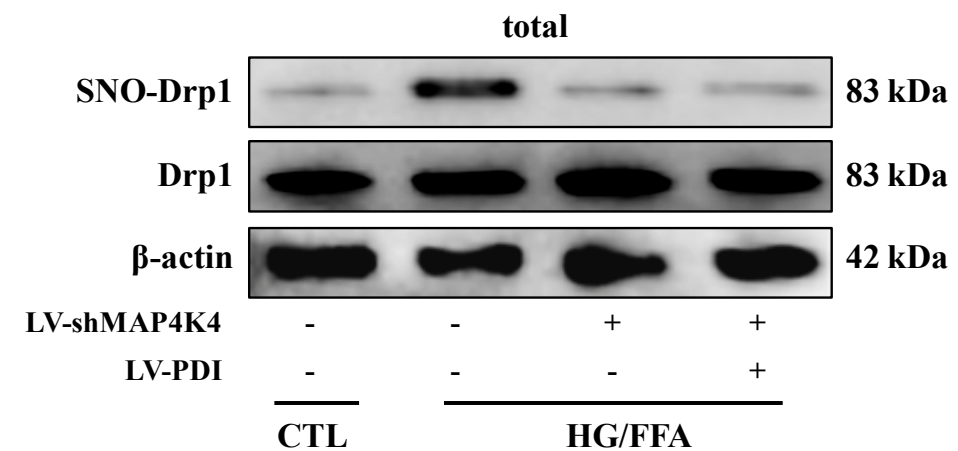**D**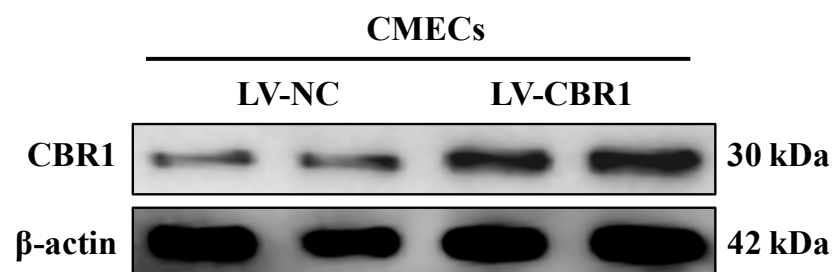**E**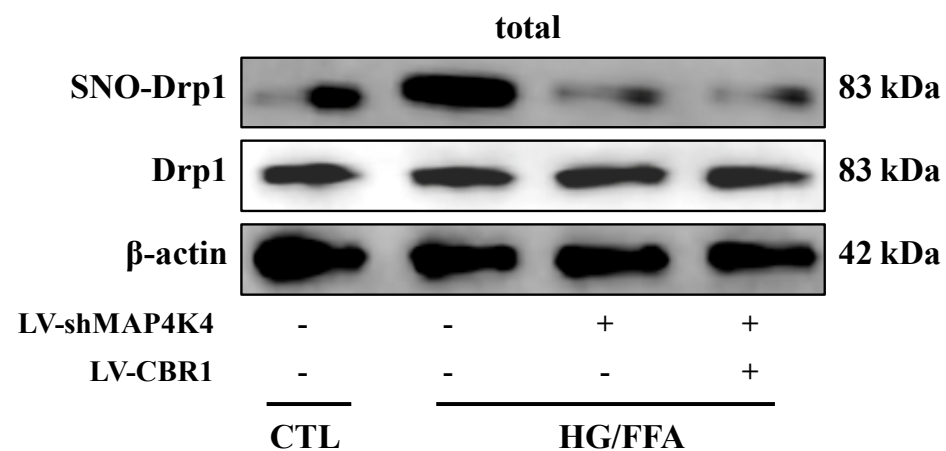**F**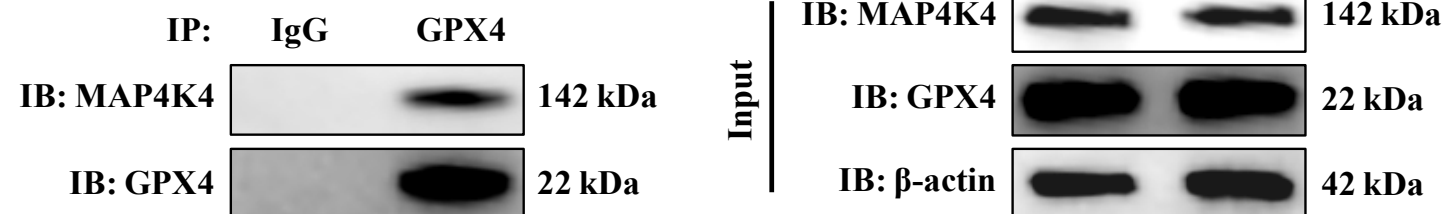**G**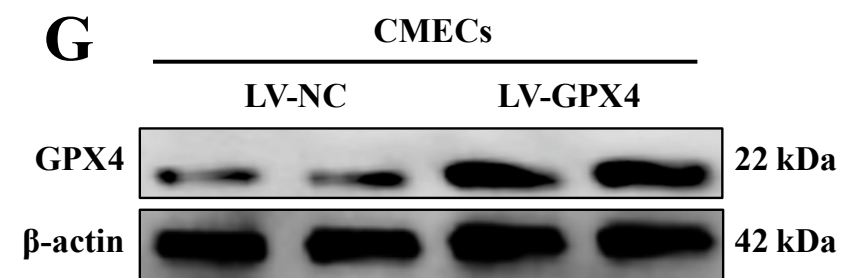**H**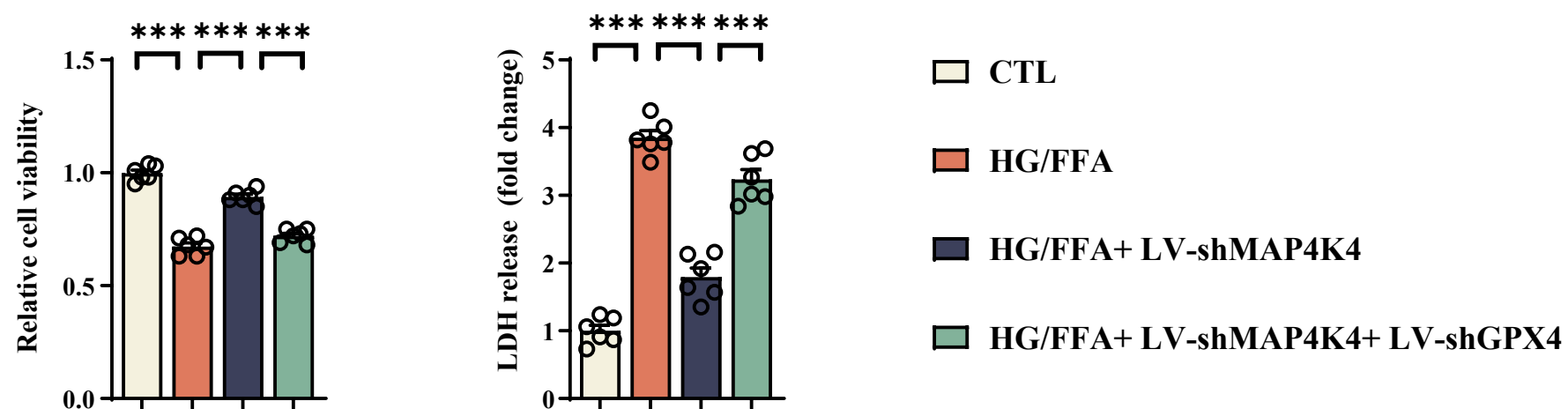

Supplement: Supplementary file 2 — Additional file 2: Figure S2. (A): The transfection efficiency of LV-shGPX4 was measured via western blotting. (B): The transfection efficiency of LV-PDI was measured via western blotting. (C): HCMECs were cotransfected with LV-shMAP4K4 and LV-PDI and subjected to HG/FFA injury. SNO-Drp1 was assessed by western blotting. (D): The transfection efficiency of LV-CBR1 was measured via western blotting. (E): HCMECs were cotransfected with LV-shMAP4K4 and LV-CBR1 and subjected to HG/FFA injury. SNO-Drp1 was assessed by western blotting. (F): A co-IP assay was carried out using an antibody against GPX4, and western blotting was performed for MAP4K4 and GPX4. (G): The transfection efficiency of LV-GPX4 was measured via western blotting. (H): HCMECs were cotransfected with LV-shMAP4K4 and LV-shGPX4 and subjected to HG/FFA injury. Relative cell viability was determined by a CCK-8 assay, and cytotoxicity was measured by an LDH release assay. *p < 0.05, **p < 0.01, ***p < 0.001 indicate significant differences. Four to six biological replicates were performed, and the results are indicated in scatter plots. [file 12933_2024_2254_MOESM2_ESM.pdf]

**A**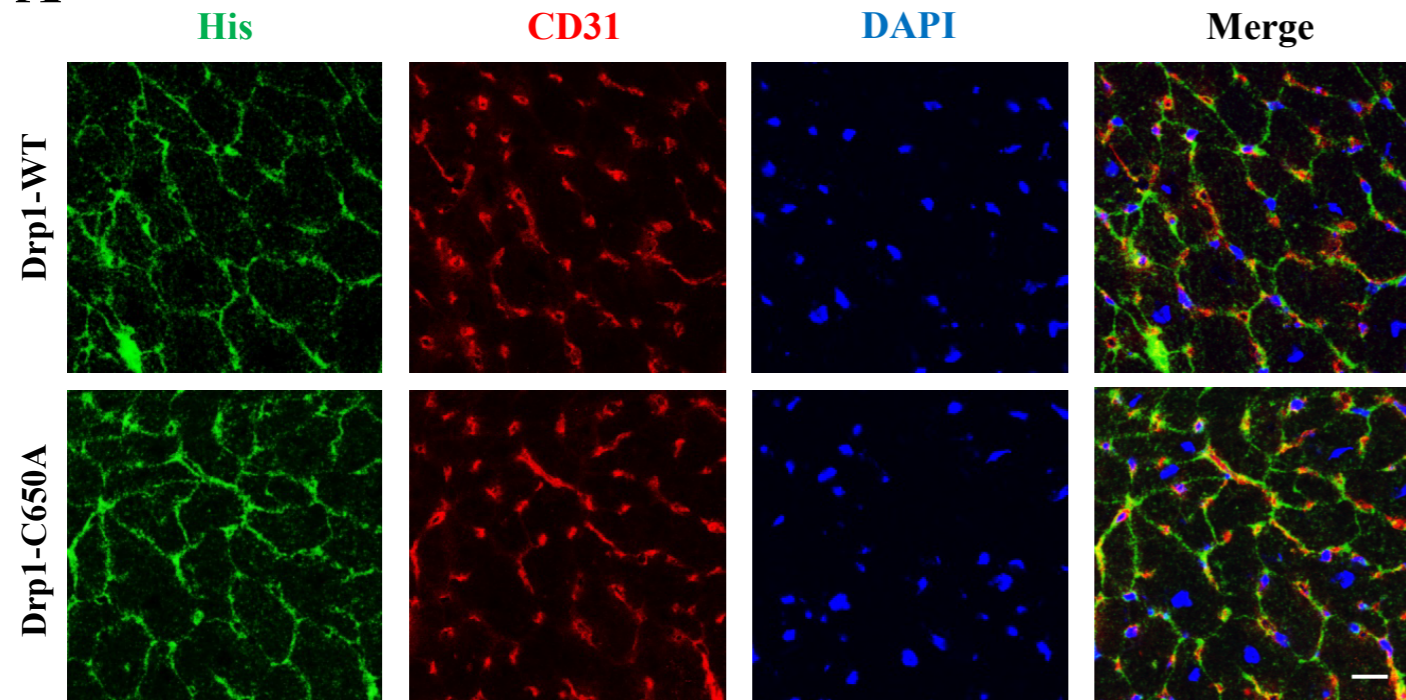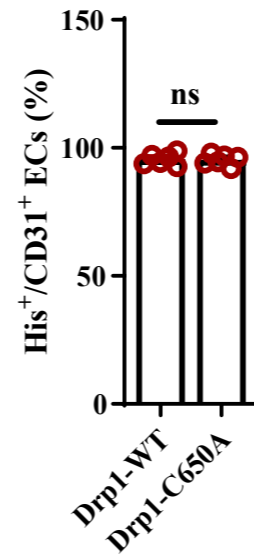**B**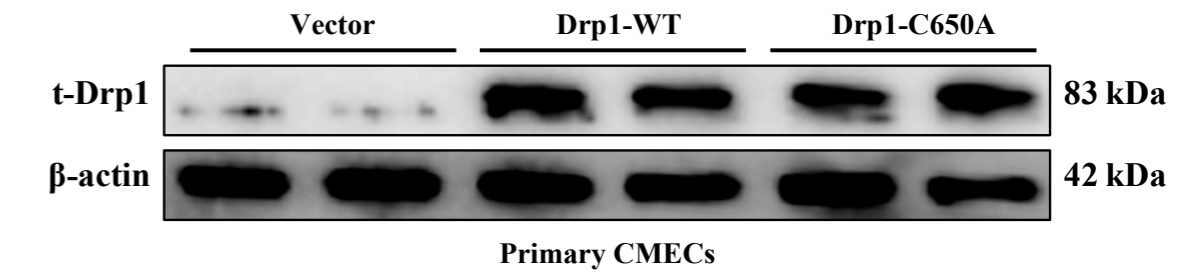**C**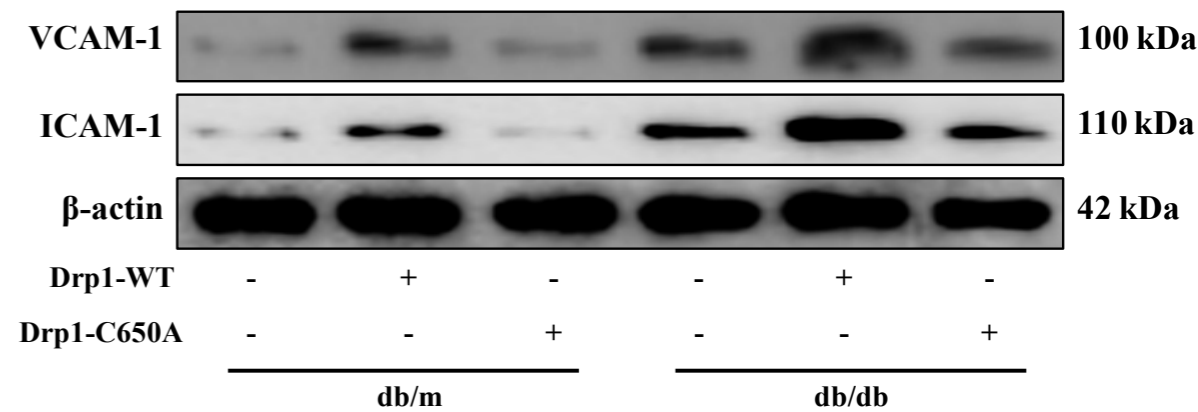**D**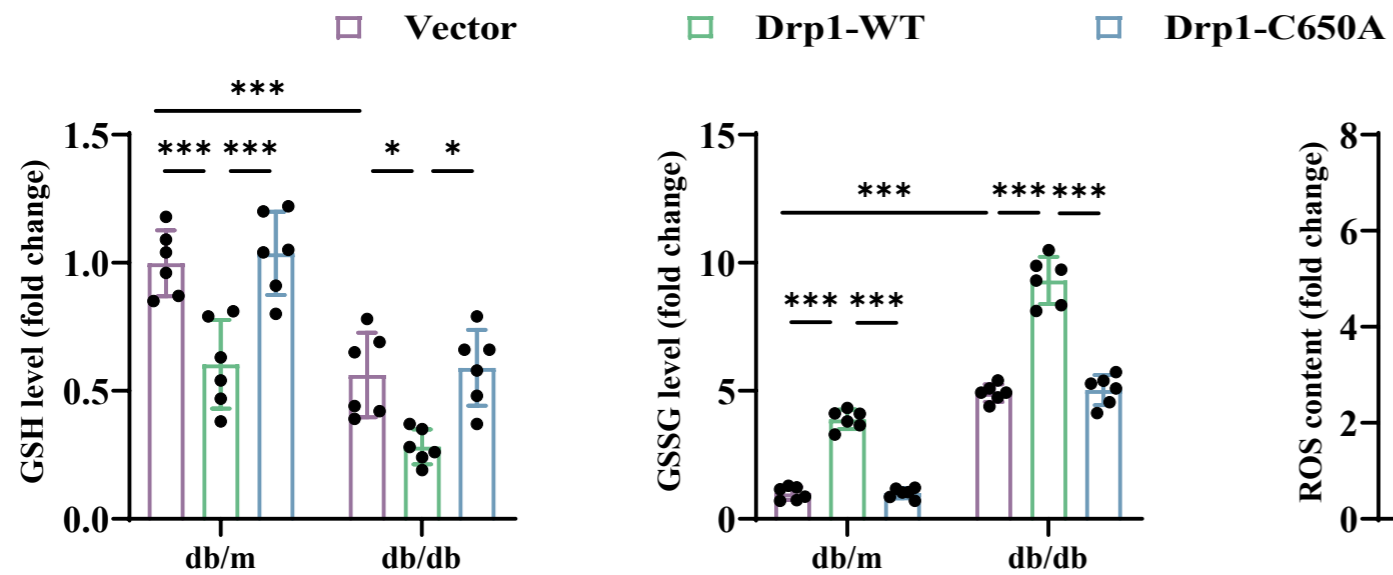**E**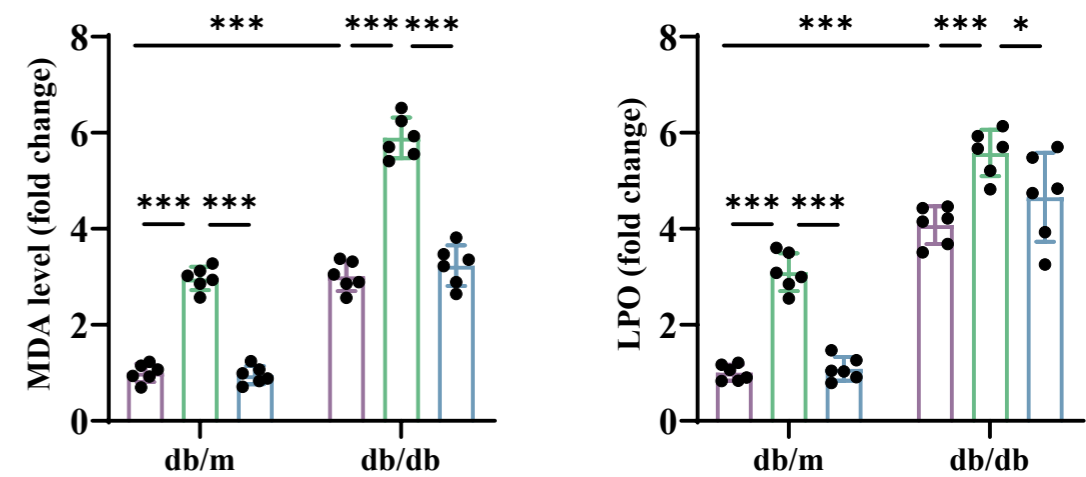

Supplement: Supplementary file 4 — Additional file 4: Figure S4. (A): Immunofluorescence staining of His-tagged AAV9 (green) and CD31-labeled CMECs (red) and statistical graphs of the percentage of His-positive CMECs. Scale bar = 50 mm. (B): The transfection efficiency of AAV9-Drp1-WT and AAV9-Drp1-C650A in primary CMECs was measured by western blotting. (C): Protein expression of VCAM-1 and ICAM-1. (D): Quantitative analysis of GSH, GSSG, and ROS levels in the indicated groups. (E): Quantitative analysis of the MDA and LPO levels in the indicated groups. *p < 0.05, **p < 0.01, ***p < 0.001 indicate significant differences. Four to six biological replicates were performed, and the results are indicated in scatter plots. [file 12933_2024_2254_MOESM4_ESM.pdf]

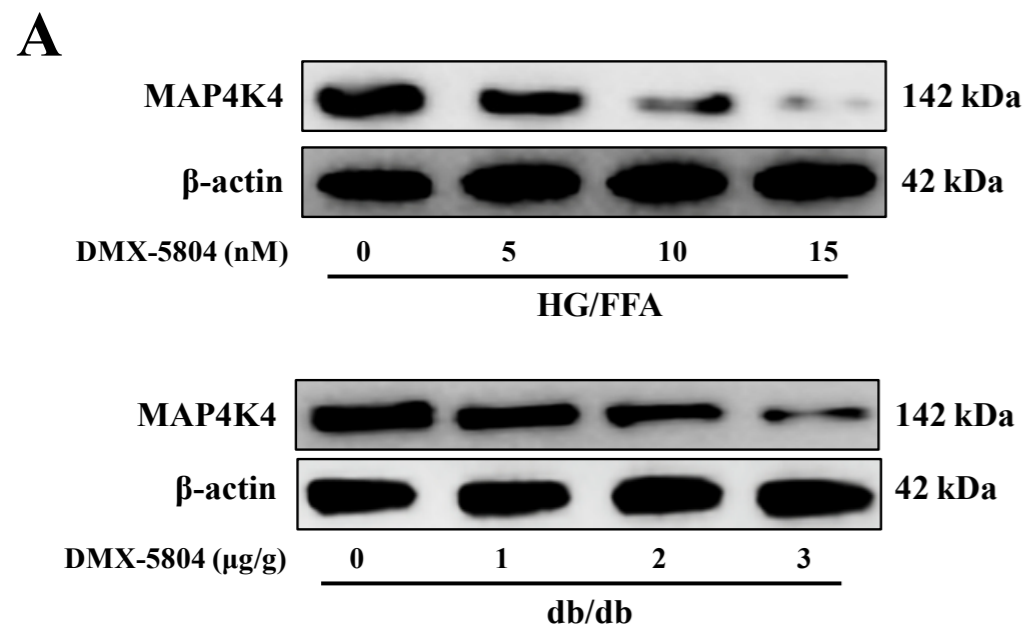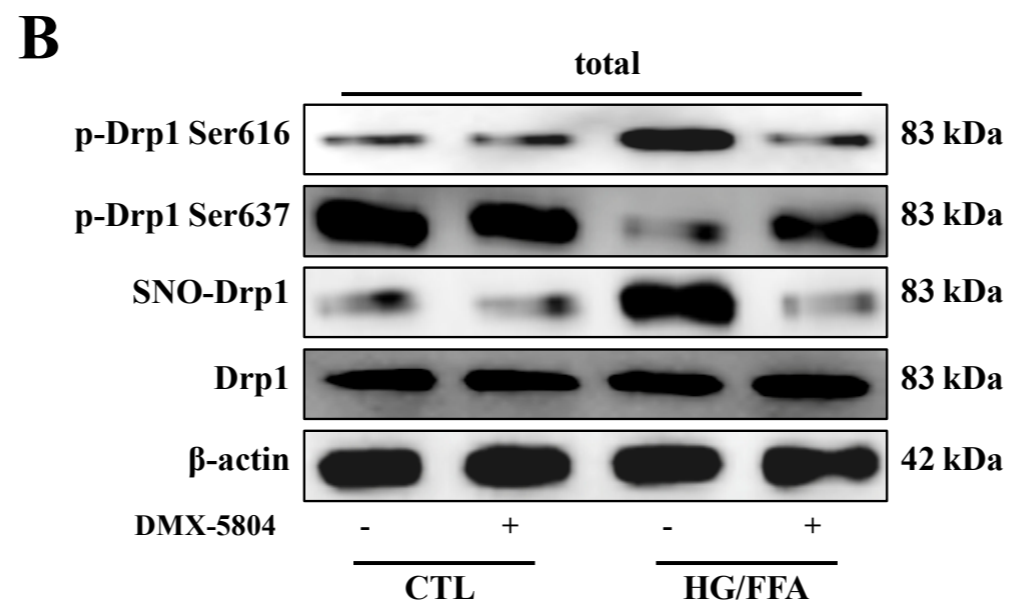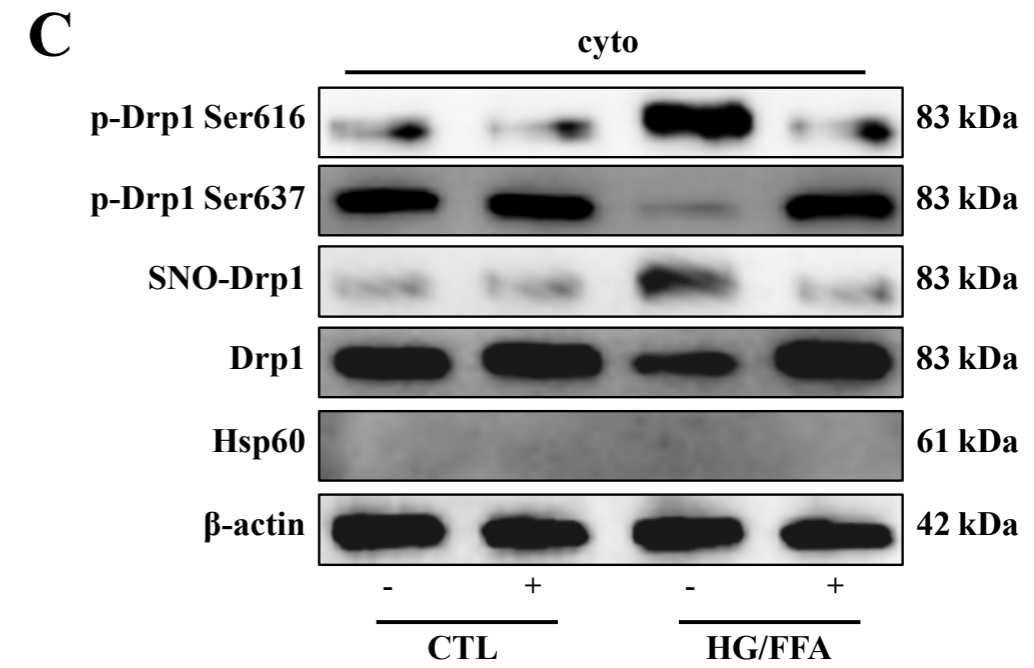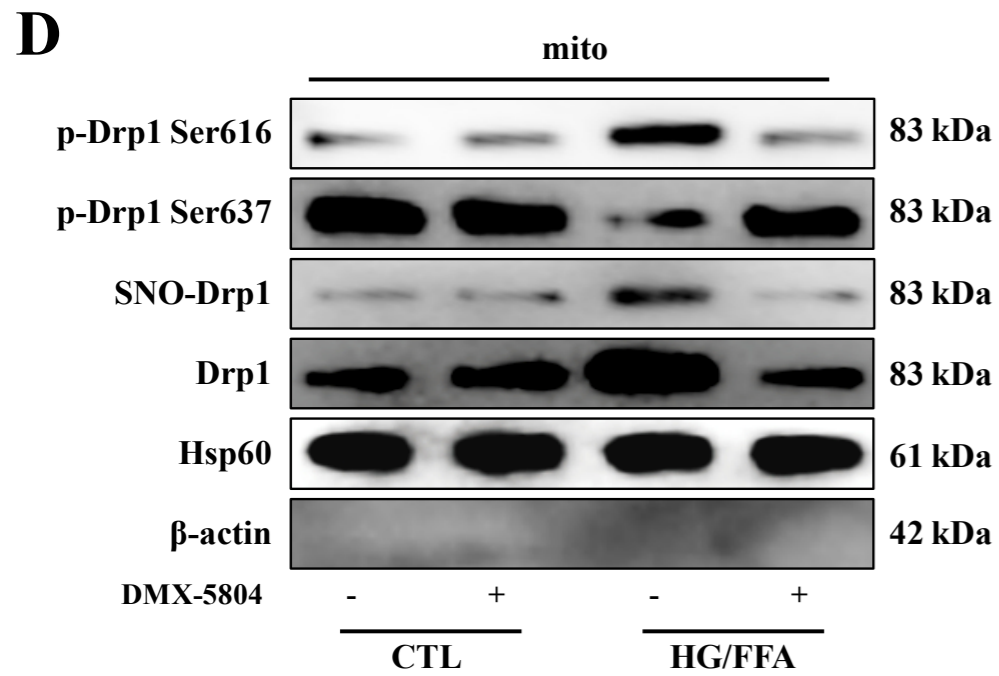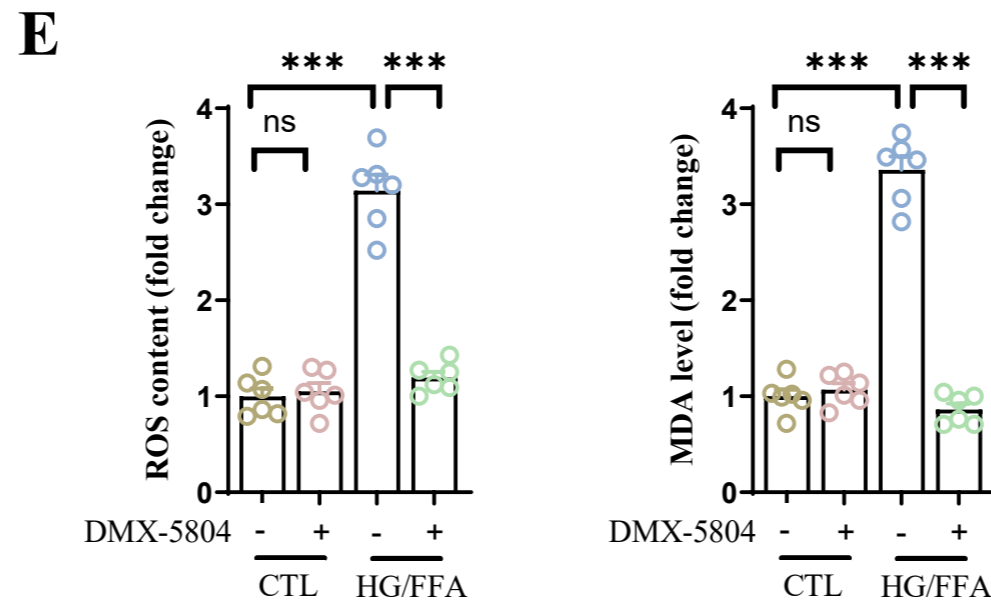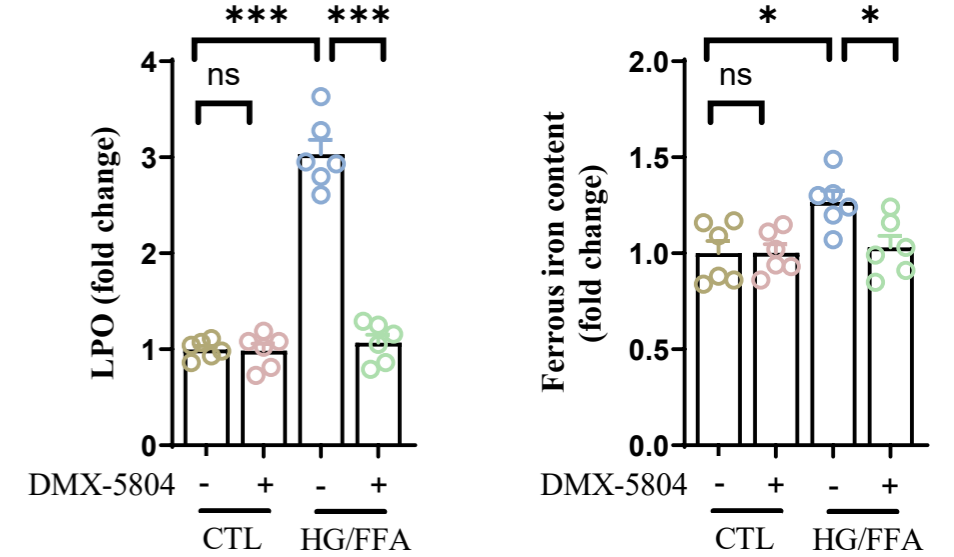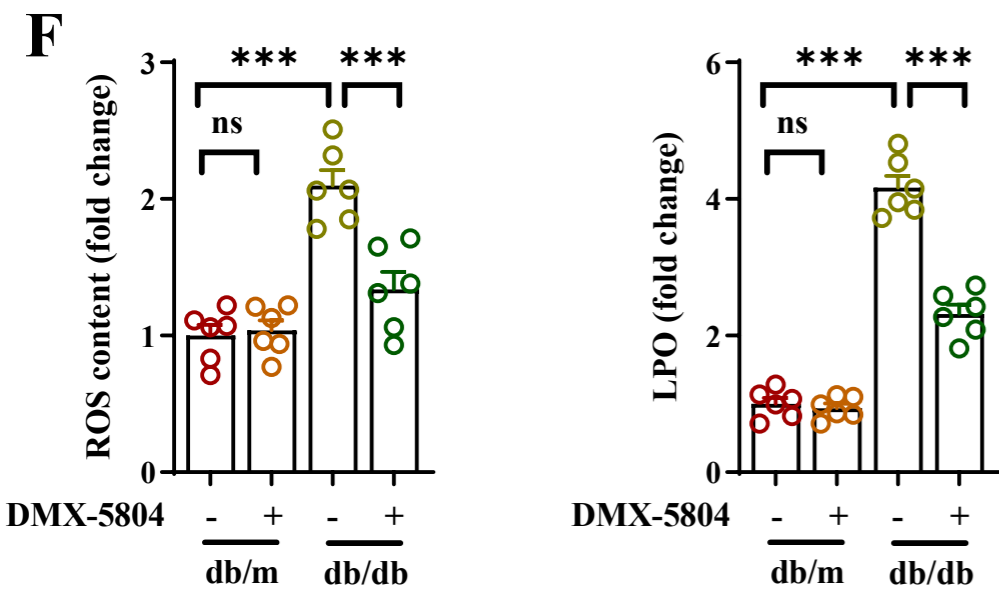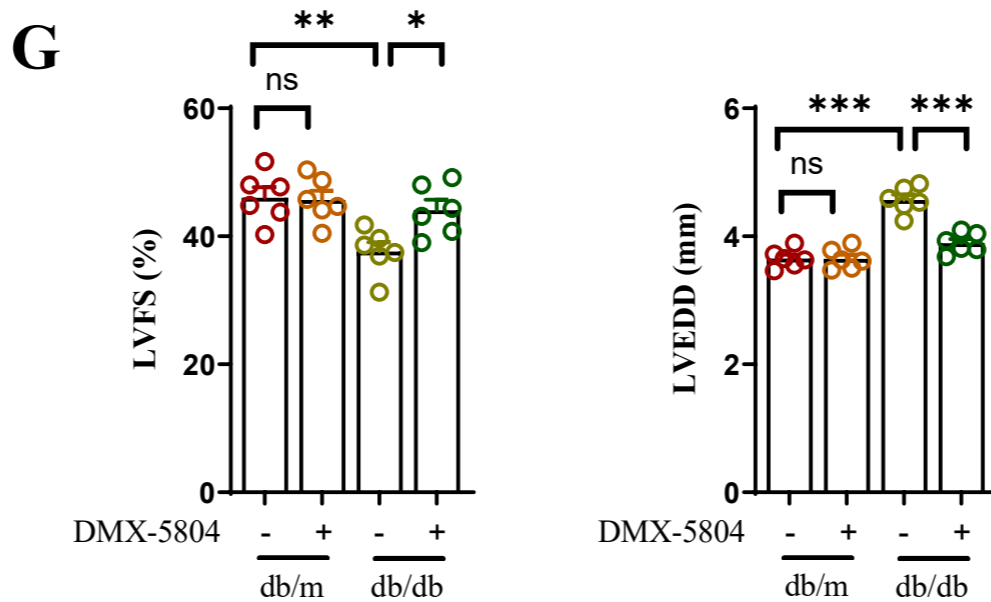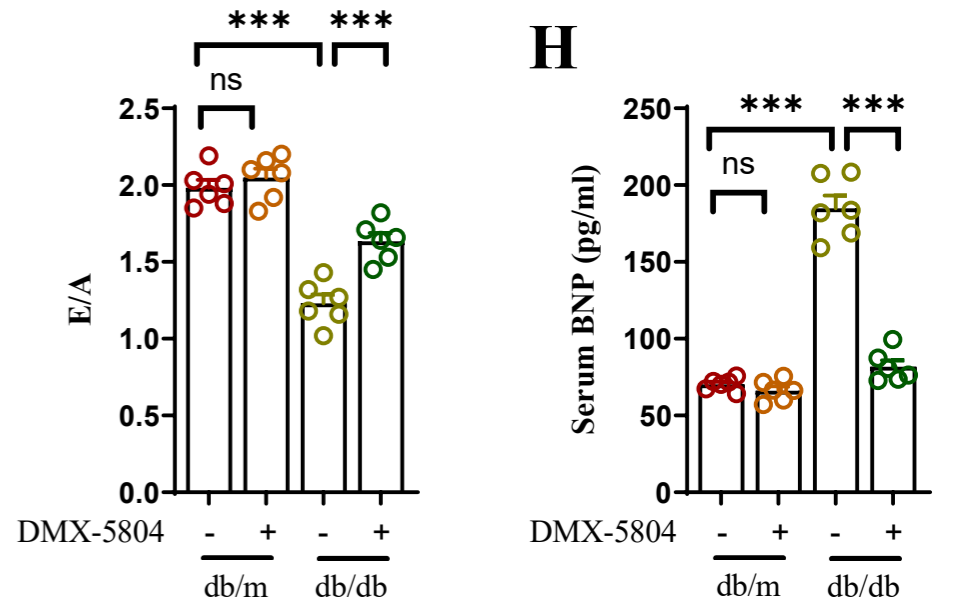

Supplement: Supplementary file 5 — Additional file 5: Figure S5. (A): Cells and db/db mice were treated with different concentrations of DMX-5804. The expression of MAP4K4 was detected by western blotting. (B): Representative immunoblotting images showing the protein expression, phosphorylation, and S-nitrosylation of Drp1 in HCMECs. (C-D): Mitochondrial and cytoplasmic levels of Drp1, Drp1 phosphorylated at Ser616, Drp1 phosphorylated at Ser637, and SNO-Drp1 were assessed by western blotting in HCMECs. (E): Quantitative analysis of ROS, MDA, LPO and ferrous iron content in HCMECs subjected to HG/FFA injury. (F): Quantitative analysis of ROS and LPO levels in cardiac tissues. (G): Statistical analysis of the LVFS, LVEDD, and E/A ratio data. (H): Quantitative analysis of serum BNP levels. *p < 0.05, **p < 0.01, ***p < 0.001 indicate significant differences. Four to six biological replicates were performed, and the results are indicated in scatter plots. [file 12933_2024_2254_MOESM5_ESM.pdf]
