# Supplementary material for: MAP4K4 exacerbates cardiac microvascular injury in diabetes by facilitating S-nitrosylation modification of Drp1
Source: Cardiovasc Diabetol. 2024 May 9;23:164. doi: 10.1186/s12933-024-02254-7 (PMC11084109; doi:10.1186/s12933-024-02254-7)
Supplement: Supplementary file 3 — Additional file 3: Figure S3. (A): Comparison of the sequence similarity of Drp1 across different species. Homo sapiens Drp1 497–514, Mus musculus Drp1 503–520, Rattus norvegicus Drp1 510–527, Bos taurus Drp1 510–527, and Danio rerio Drp1 496–513. The Drp1 C505 site in humans is highly conserved across different species. (B): Comparison of the sequence similarity of Drp1 across different species. Homo sapiens Drp1 636–653, Mus musculus Drp1 642–659, Rattus norvegicus Drp1 655–672, Bos taurus Drp1 649–666, and Danio rerio Drp1 591–608. The Drp1 C644 site in humans and the C650 site in mice are highly conserved across different species. (C): Genomic DNA was extracted from the indicated cells with or without C505A knockdown. PCR products were amplified and sequenced. (D): Genomic DNA was extracted from the indicated cells with or without C644A knockdown. PCR products were amplified and sequenced. (E): The transfection efficiency of LV-MAP4K4 was measured via western blotting. (F): Quantitative analysis of H2O2 content in the indicated groups. (G): Statistical analysis of the number of migrated cells in the Transwell assay. (H): Statistical analysis of NO release. *p < 0.05, **p < 0.01, ***p < 0.001 indicate significant differences. Four to six biological replicates were performed, and the results are indicated in scatter plots. [file 12933_2024_2254_MOESM3_ESM.pdf]

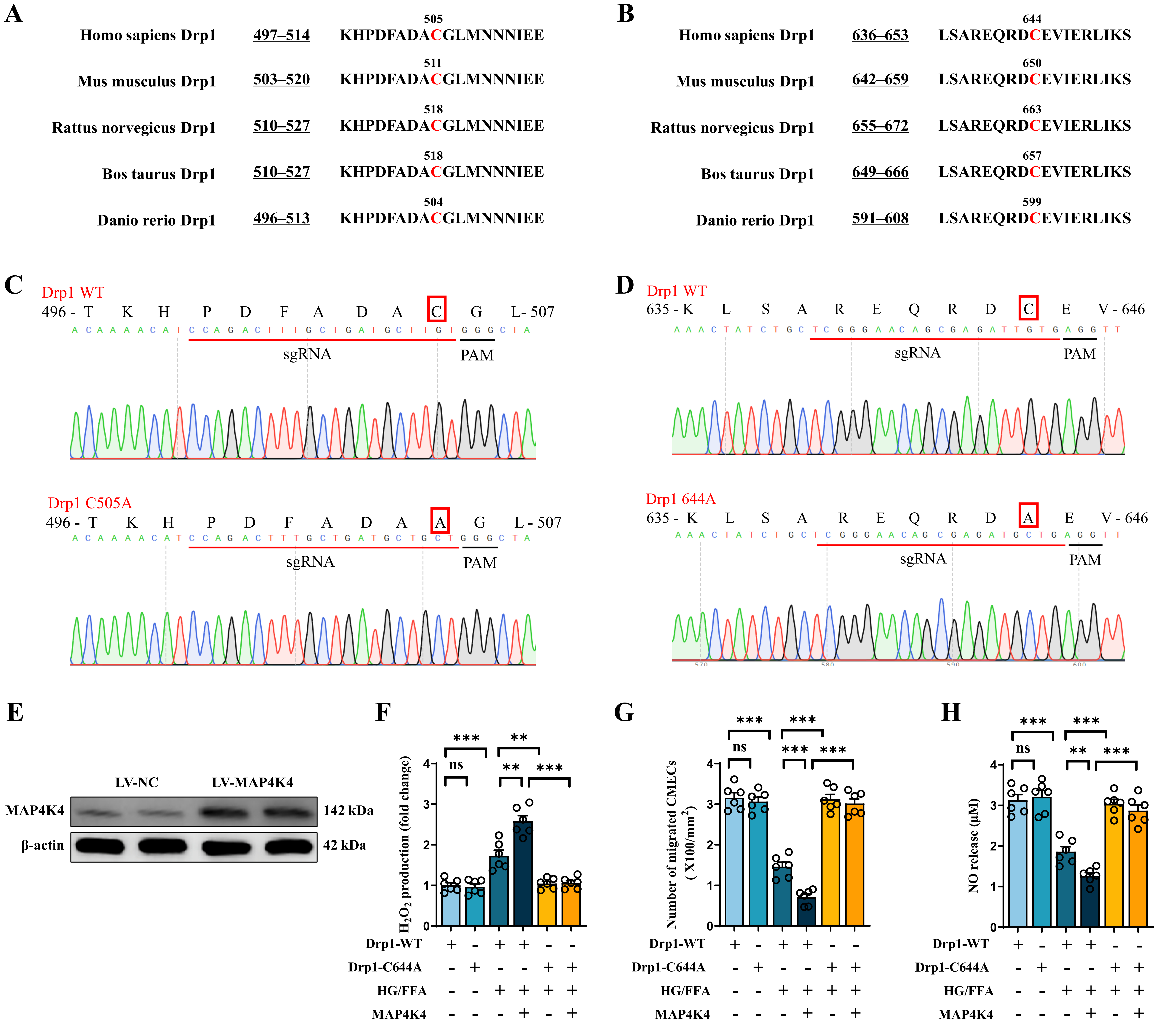
A
Homo sapiens Drp1 497–514 505 KHPDFADACGLMNNNIEE
Mus musculus Drp1 503–520 511 KHPDFADACGLMNNNIEE
Rattus norvegicus Drp1 510–527 518 KHPDFADACGLMNNNIEE
Bos taurus Drp1 510–527 518 KHPDFADACGLMNNNIEE
Danio rerio Drp1 496–513 504 KHPDFADACGLMNNNIEE
B
Homo sapiens Drp1 636–653 644 LSAREQRDCEVIERLIKS
Mus musculus Drp1 642–659 650 LSAREQRDCEVIERLIKS
Rattus norvegicus Drp1 655–672 663 LSAREQRDCEVIERLIKS
Bos taurus Drp1 649–666 657 LSAREQRDCEVIERLIKS
Danio rerio Drp1 591–608 599 LSAREQRDCEVIERLIKS
C
Drp1 WT
496 - T K H P D F A D A C G L- 507
sgRNA
PAM
Drp1 C505A
496 - T K H P D F A D A A G L - 507
sgRNA
PAM
D
Drp1 WT
635 - K L S A R E Q R D C E V - 646
sgRNA
PAM
Drp1 644A
635 - K L S A R E Q R D A E V - 646
sgRNA
PAM
E
LV-NC
LV-MAP4K4
MAP4K4
142 kDa
β-actin
42 kDa
F
H2O2 production (fold change)
G
Number of migrated CMECs (X100/mm2)
H
NO release (μM)
ns
**
***
Drp1-WT
Drp1-C644A
HG/FFA
MAP4K4
