# Supplementary material for: MAP4K4 exacerbates cardiac microvascular injury in diabetes by facilitating S-nitrosylation modification of Drp1
Source: Cardiovasc Diabetol. 2024 May 9;23:164. doi: 10.1186/s12933-024-02254-7 (PMC11084109; doi:10.1186/s12933-024-02254-7)
Supplement: Supplementary file 6 — Additional file 6: Table S1 Primer. Table S2 Primary antibodies used in western blots. [file 12933_2024_2254_MOESM6_ESM.pdf]

Supplementary Table 1	
DRP1	Forward Primer CTGCCTCAAATCGTCGTAGTG
	Reverse Primer GAGGTCTCCGGGTGACAATTC
Fis1	Forward Primer GATGACATCCGTAAAGGCATCG
	Reverse Primer AGAAGACGTAATCCCGCTGTT
Dcn	Forward Primer ATGAAGGCCACTATCATCCTCC
	Reverse Primer GTCGCGGTCATCAGGAACTT
Mapt	Forward Primer CCAAGTGTGGCTCATTAGGCA
	Reverse Primer CCAATCTTCGACTGGACTCTGT
Pink1	Forward Primer GGAGGAGTATCTGATAGGGCAG
	Reverse Primer AACCCGGTGCTCTTTGTCAC
Bnip3	Forward Primer CAGGGCTCCTGGGTAGAACT
	Reverse Primer CTACTCCGTCCAGACTCATGC
Mief1	Forward Primer CACGGCCATTGACTTTGTGC
	Reverse Primer TCGTACATCCGCTTAACTGCC
Mief2	Forward Primer ATGGCAGAGTTCTCCCAGAAA
	Reverse Primer CCCTGTCAATGAACCGCTT
Mff	Forward Primer ACTGAAGGCATTAGTCAGCGA
	Reverse Primer TCCTGCTACAACAATCCTCTCC
Ggnbp1	Forward Primer TCTTTGAACTGGCTTACCACG
	Reverse Primer GCAGGATTTCACGTTGCTCC

Senp5	Forward Primer GGGAAGGCCAGTTACTTGGAA
	Reverse Primer CAAAGGGGTTCATCCTTGATCC
Ddhd2	Forward Primer ATGTCATCAGTGCAGTCACAAC
	Reverse Primer ACTGGTTCATACAAGCTGCCA
Mtfp1	Forward Primer GTGTGCTGCCTCTCTCTATGT
	Reverse Primer TGGGGTGGATAATGATGGGGA
Mtfr2	Forward Primer AATGACCTGCCTGTAAATGAAGC
	Reverse Primer TGCAATCTGAGAGCGAAGAAAAG
Prkn	Forward Primer CCCACCTCTGACAAGGAAACA
	Reverse Primer TCGTGAACAAACTGCCGATCA
Miga2	Forward Primer GAAGCAGGTTGGTCCCGAG
	Reverse Primer ACTCAGGGTGTCGTTGCTCT
Mfn1	Forward Primer GAGGTGCTATCTCGGAGACAC
	Reverse Primer GCCAATCCCACTAGGGAGAAC
Mfn2	Forward Primer GGCCCAACTCTAAGTGCCC
	Reverse Primer AAGTGCTTTTCCGTCTGCATC
Opa1	Forward Primer TGTGAGGTCTGCCAGTCTTTA
	Reverse Primer TGTCCTTAATTGGGGTCGTTG
β-actin	Forward Primer CATGTACGTTGCTATCCAGGC
	Reverse Primer CTCCTTAATGTCACGCACGAT

Table S2 Primary antibodies used in western blots.

Name	Manufacturer	Cat No.	Dilution	Reacts with:
β-actin	Abcam	Ab8226	1:1000	Mouse, Rat, Human
MAP4K4	Abcam	Ab80418	1:1000	Mouse, Rat, Human
BNIP3	Abcam	Ab109362	1:1000	Mouse, Rat, Human
GPX4	Abcam	Ab125066	1:3000	Mouse, Rat, Human
CD31	Abcam	Ab281583	1:1000	mice, human
eNOS	Abcam	Ab76198	1:1000	Mouse, Human
eNOS (phospho S1177)	Abcam	Ab230158	1:1000	Mouse, Human
VEGFR2	Abcam	Ab11939	1:1000	Mouse, Rat, Human, Recombinant fragment
VEGFR2 (Tyr1175)	CST	2478	1:2000	mice, human
ICAM-1	Affinity	AF6088	1:1000	mice, human
VCAM-1	Abcam	Ab134047	1:2000	mice, human
Drp1	Abcam	Ab184247	1:1000	Mouse, Rat, Human
Drp1(Ser616)	Bioss	Bs-12702R	1:300	mice
DRP1 (phospho S637)	Abcam	Ab193216	1:1000	Mouse, Rat, Human
MFN1	Abcam	Ab126575	1:200	Mouse, Rat
VDAC1	Abcam	Ab154856	1:1000	Mouse, Rat, Human
MIEF1	Affinity	DF12019	1:1000	Mouse, Rat, Human
DHODH	Abcam	Ab246901	1:1000	Mouse, Rat, Human
GSNOR	Abcam	Ab175406	1:1000	Mouse, Rat, Human
TRX	Abcam	Ab273877	1:1000	Mouse, Rat
TRX2	Abcam	Ab185544	1:10000	Mouse, Human
GLRX1	Abcam	Ab45953	1:250	Mouse, Rat, Human
PDI	Affinity	DF7593	1:1000	Mouse, Rat, Human
GPX1	Abcam	Ab22604	1:1000	Mouse, Rat, Human
GPX3	Abcam	Ab256470	1:1000	Mouse, Rat
CBR1	Abcam	Ab186825	1:1000	Mouse, Human
CBR3	Abcam	Ab180939	1:10000	Mouse, Rat, Human
